# Unveiling the Prevalence and Surgical Burden of Neurocristopathies: A Scoping Review and Statistical Analysis

**DOI:** 10.21203/rs.3.rs-10318477/v1

**Published:** 2026-08-06

**Authors:** Bryan S. Torres, Caitlin Foster, Shaun R. Abrams, Laura Kerosuo

**Affiliations:** 1. Neural Crest Development and Disease Unit, National Institute of Dental and Craniofacial Research, National Institutes of Health, Bethesda, Maryland, USA.; 2. Craniofacial Development Unit, National Institute of Dental and Craniofacial Research, National Institute of Health, Bethesda, MD, USA.; 3. Tulane University, School of Medicine, New Orleans, LA, USA.; 4. Division of Plastic and Reconstructive Surgery, University of Colorado Anschutz Medical Campus, Aurora, CO, USA

## Abstract

**Background:**

The prevalence, clinical burden, impact on quality of life, and multidisciplinary treatment planning of Neurocristopathies (NCPs), disorders arising from aberrant development of neural crest cells (NCCs), remain obscure. NCCs are a pleistopotent embryonic stem cell population with diverse progeny of cell types ranging from craniofacial skeleton to cardiac outflow tract, peripheral nervous system, chromaffin cells, melanocytes etc. Given the broad range of neural crest derivatives, patients often present with complex, multisystem manifestations. However, these diverse symptoms may be overlooked, resulting in surgical planning that addresses only a single manifestation at a time rather than integrating the patient’s full clinical presentation into treatment decisions.

**Methods:**

This scoping review with statistical analysis identified 92 distinct NCPs to quantify prevalence, anatomical defect patterns, cell type of origin and surgical management needs across craniofacial and non-craniofacial phenotypes.

**Results:**

When scaled to global birth statistics, NCPs accounted for ~4.24% of all live births, indicating that more than half (61%) of serious congenital anomalies are neurocristopathy in origin, greatly exceeding prior estimates. These primarily consisted of craniofacial, conotruncal, and peripheral nervous system defects. Plastic and reconstructive surgery (PRS) (which significantly overlap in procedures of Oral & Maxillofacial Surgery in the Craniofacial complex) emerged as a central clinical modality: 61 NCPs (66.3%) required PRS/OMFS, and nearly 100% of craniofacial bone and cartilage anomalies required functional reconstruction. Mandibular reconstruction (37%) and cleft lip/palate repair (37%) represented the most common interventions, with midface advancement, ear reconstruction, and cranial vault remodeling also frequently required. Chi-square analysis demonstrated a strong association between anatomical category and likelihood of PRS intervention (χ^2^ = 52.91, p < 0.001), with craniofacial involvement conferring the highest risk.

**Conclusions:**

These findings establish NCPs as a major, underrecognized driver of congenital disease burden and demonstrate the critical role of PRS in restoring function and form across neurocristopathy phenotypes.

## Background

**Figure1:**
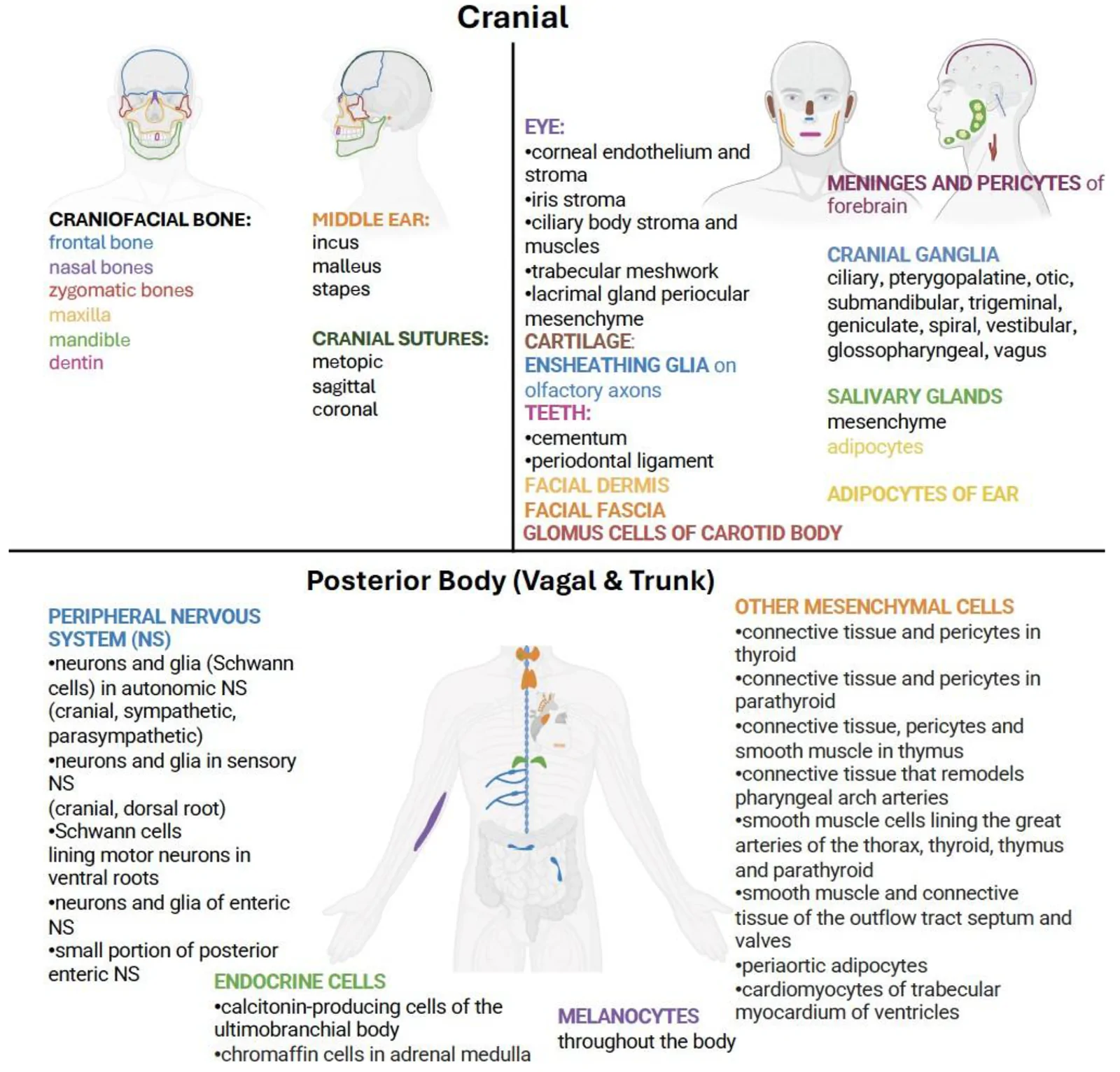
Neural crest cell derivatives and anatomical basis for neurocristopathy categorization. The cartoons illustrate the broad developmental contributions of neural crest cells (NCCs) across craniofacial and posterior body structures, providing the developmental biology framework for neurocristopathy (NCP) etiology and classification used in this review. The derivatives are divided into cranial and posterior body (which includes both vagal and trunk axial level) counterparts. Together, these neural crest-derived structures underpin the four major disease categories analyzed in this study: neurocristopathies that cause defects in craniofacial bone and cartilage (CFS B&C), craniofacial non-skeletal structures (CFS Other), posterior body, and syndromic including those which affect both craniofacial and posterior body regions (shown in Figure 2). The figure is modified from (1) which was complemented with (131–133).

Neural crest cells (**NCCs**) represent a remarkable, highly migratory, and pluripotent-like “pleistopotent” stem cell population unique to vertebrate embryonic development (1, 2). Originating from the ectodermal germ layer at the neural plate border during neurulation, NCCs are specified in the dorsal neural tube and go through a process known as epithelial-to-mesenchymal transition (EMT) to gain complex migratory properties that allow them to differentiate to a wide array of cell types throughout the body. These include typical ectodermal cells like neurons and glia of the peripheral nervous system and melanocytes, but also atypical derivatives like osteoblasts and chondrocytes, cardiac outflow tract connective tissue, pericytes, smooth muscle and endocrine cells, to name a few (see full derivative list in Figure 1), which typically are formed from the mesoderm and endoderm germ layers^1^. This developmental plasticity and exceptionally broad differentiation potential of NCCs to contribute to cell types beyond the rule of gastrulation has led researchers to regard them as a “fourth germ layer” (1).

Neural crest cells (NCCs) play vital roles in embryonic development, making them vulnerable to disruptions that give rise to a diverse group of congenital disorders known as neurocristopathies (NCPs). These disorders result from defects in NCC specification, migration, differentiation, or survival, and manifest across multiple tissues. Clinical features include craniofacial anomalies (e.g., cleft lip and palate, mandibular hypoplasia, craniosynostosis), cardiac outflow tract defects (e.g., conotruncal anomalies), enteric nervous system dysfunction (e.g., Hirschsprung disease), and pigmentation abnormalities (e.g., piebaldism, neurocutaneous syndromes, Waardenburg syndrome). Craniofacial conditions such as cleft lip and palate (CLP), a common phenotype globally, and Treacher Collins syndrome (TCS), a rarer but more severe disorder characterized by facial bone and soft tissue underdevelopment, can significantly impair mastication, feeding, breathing, speech, and psychosocial development of self-esteem. Hirschsprung disease often presents with aganglionic colon, leading to intestinal obstruction. The pleistopotency of NCCs is reflected in the multifaceted nature of defects in syndromes where several NCC derived cell types are defected, like in Waardenburg where pigmentation disorders may be associated with sensorineural deafness and craniofacial anomalies. Similarly, DiGeorge syndrome (22q11.2 deletion) presents with a combination of craniofacial anomalies (e.g., cleft palate, micrognathia), congenital heart defects (e.g., interrupted aortic arch, tetralogy of Fallot), thymic hypoplasia, and parathyroid dysfunction (3–10). These patients frequently require coordinated, multidisciplinary care, including cardiac, craniofacial, and immunologic surgical interventions. The range and severity of these manifestations highlight the substantial impact of NCPs on health, functional ability, and quality of life, reinforcing the need for early diagnosis, integrated management, and long-term support.

Congenital anomalies represent a significant health burden, affecting approximately 1 in 33 infants in the United States, according to the National Institute of Child Health and Human Development and the Centers for Disease Control and Prevention (11–13). Birth defects are the leading cause of infant deaths and rank as the second and third leading causes of death in children aged 1–4 and 5–9 years, respectively (14). On a global scale, the World Health Organization estimates that 6–7% of infants are born with congenital disorders, excluding terminated pregnancies and stillbirths, emphasizing the profound impact of these anomalies on pediatric morbidity and mortality (15).

Historically, 15–30% of all birth defects are mentioned as attributable to NCPs (3, 16–18), yet these estimates are vague and not based on rigorous validation of any type of credible data. Given the critical developmental role of NCCs and their contribution to diverse structures, research into NCP prevalence and surgical implications is essential to improve early diagnosis and guide multidisciplinary treatment planning. Despite their clinical impact, NCPs remain underrecognized, particularly in the field of plastic and reconstructive surgery (**PRS**). The varied clinical presentations resulting from NCC-derived anomalies often necessitate a multidisciplinary team, including plastic surgeons, oral and maxillofacial surgeons, dentists, orthodontists, speech-language pathologists, developmental biologist, geneticists, and other allied specialists, each contributing to the complex needs of affected patients (19). Plastic surgeons address the complex reconstructive challenges posed by NCPs, improving functional and psychosocial outcomes. This study provides a comprehensive analysis of NCP prevalence, cell/tissue type of origin and surgical needs, particularly craniofacial involvement, categorizing defect types and distinguishing between functional and aesthetic surgical requirements. This work aims to clarify PRS’s role in managing NCP-related anomalies, optimize surgical outcomes, and inform future neural crest research.

## Results

### 92 neurocristopathies were identified and analyzed for the study

**Figure 2:**
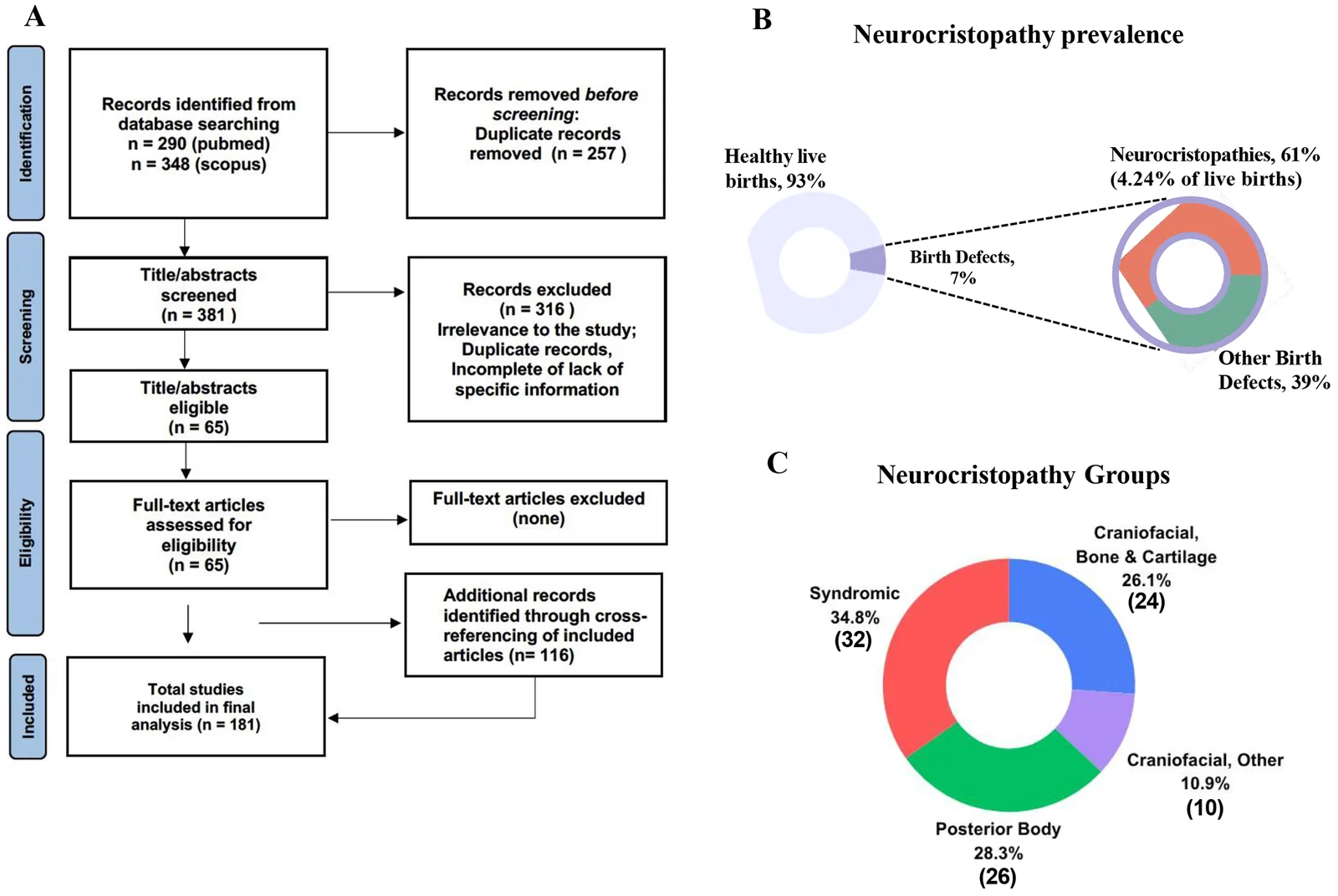
Identification, prevalence, and anatomical distribution of neurocristopathies. **(A)** Flow diagram depicting study identification, screening, eligibility assessment, and inclusion. A total of 638 records were identified through database searching, with 181 articles ultimately included following duplicate removal, abstract and full-text screening, and targeted manual cross-referencing of selected articles with particular focus on high-yield reviews. **(B)** Proportional contribution of neurocristopathies to congenital anomalies shows that they accounted for approximately 4.24% of all live births, corresponding to ~61–65% of birth defects, based on global estimates that 6–7% of live births are affected by congenital anomalies. **(C)** Distribution of identified neurocristopathies by anatomical category. Conditions were classified into craniofacial bone and cartilage (CFS B&C), craniofacial non-skeletal structures (Craniofacial, Other), posterior body and Syndromic (combination of both craniofacial and posterior body regions) neurocristopathies, reflecting the diverse developmental contributions of neural crest cells across the craniofacial complex and posterior body axis.

**Figure 3:**
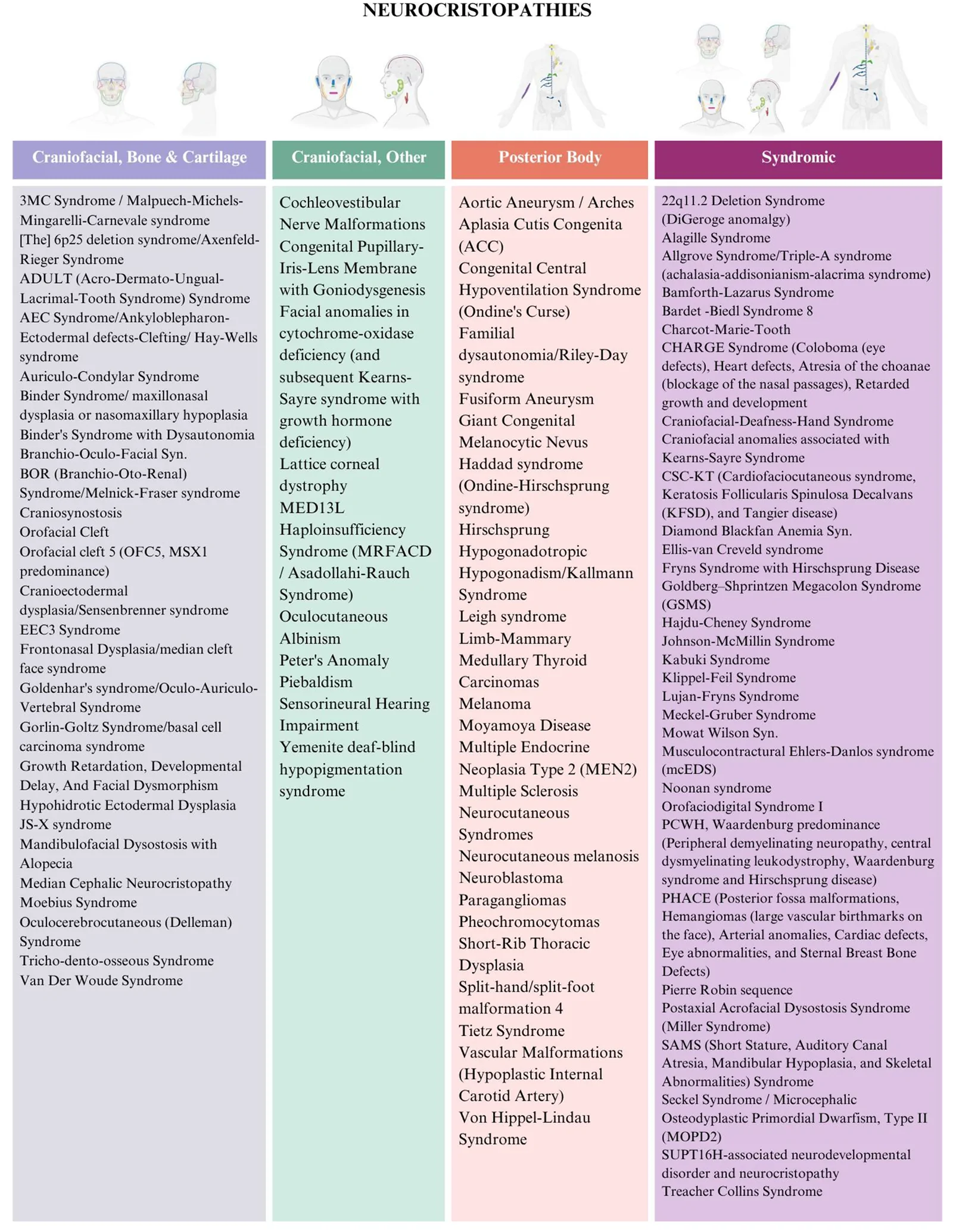
Comprehensive classification of identified neurocristopathies. A tabulation of 92 neurocristopathies that were identified from the 181 studies included in this work are summarized by anatomical category (Craniofacial Bone and Cartilage, Craniofacial Other, Posterior Body, and Syndromic). Syndromic category includes neurocristopathies with phenotypes including both anterior and posterior anatomical categories. Additional details of respective defect type, associated surgical interven tions, and key clinical features with full references and detailed annotations are provided in the Supplementary Data Table 1.

A total of 638 records were initially identified through database searching (290 records from pubmed and 348 records from scopus). After removing 257 duplicates, 381 titles and abstracts were screened. Of these, 316 were excluded due to irrelevance, insufficient detail on neurocristopathies (NCPs), non-human data, or non-English language articles that could not be interpreted. This resulted in 65 articles selected for full-text review. Following full-text assessment, the 65 articles for full-text review were verified as meeting all inclusion criteria. To ensure a comprehensive and accurate assessment of neurocristopathies (NCPs), we supplemented our database-derived articles with a targeted manual cross-referencing strategy. After identifying 65 studies that met full-text inclusion criteria, we examined the bibliographies of these articles, especially high-yield reviews such as Vega et al. (20) and Chatzi et al. (21), to locate original or primary publications on NCPs that were not captured in the initial search. This deliberate approach allowed us to identify 116 additional studies, enhancing the completeness of our dataset for prevalence estimation and surgical classification. In total, 181 articles were included in the final analysis (Figure 2A). We identified 92 NCPs (Figure 3, Supplemental Table 1) with detailed information on prevalence, anatomical classification (craniofacial bone and cartilage, other craniofacial, posterior body, and syndromic), defect types (bone, cartilage, mixed, indirectly affected, or neither), as well as plastic vs. non-plastic surgery interventions associated with the respective disease. Additionally, to characterize clinical variability, metrics such as age of onset, life expectancy, and surgical specifics (functional, aesthetic, or mixed) were incorporated, and everything was pooled into a comprehensive table together with the information of the respective references used (Supplemental Table 2).

### Over 50% of birth defects consist of neurocristopathies

The cumulative prevalence of neurocristopathies (NCPs), when scaled to a per-million metric, was approximately 4.24% of all live births (or 42 in a thousand), indicating that ~65% of birth defects (6-7% of all live births) are NCPs (Figure 2B). When compared to global estimates that 6% of live births have serious congenital anomalies, our analysis suggests that NCPs account for 61% of these conditions. In sum, our results indicate that at least 50% of birth defects are NCPs thus substantially exceeding previous hear-say based estimates of 15–30%.

### Defect Type Distribution and Surgical Intervention Patterns Across Neurocristopathy Subgroups

**Figure 4:**
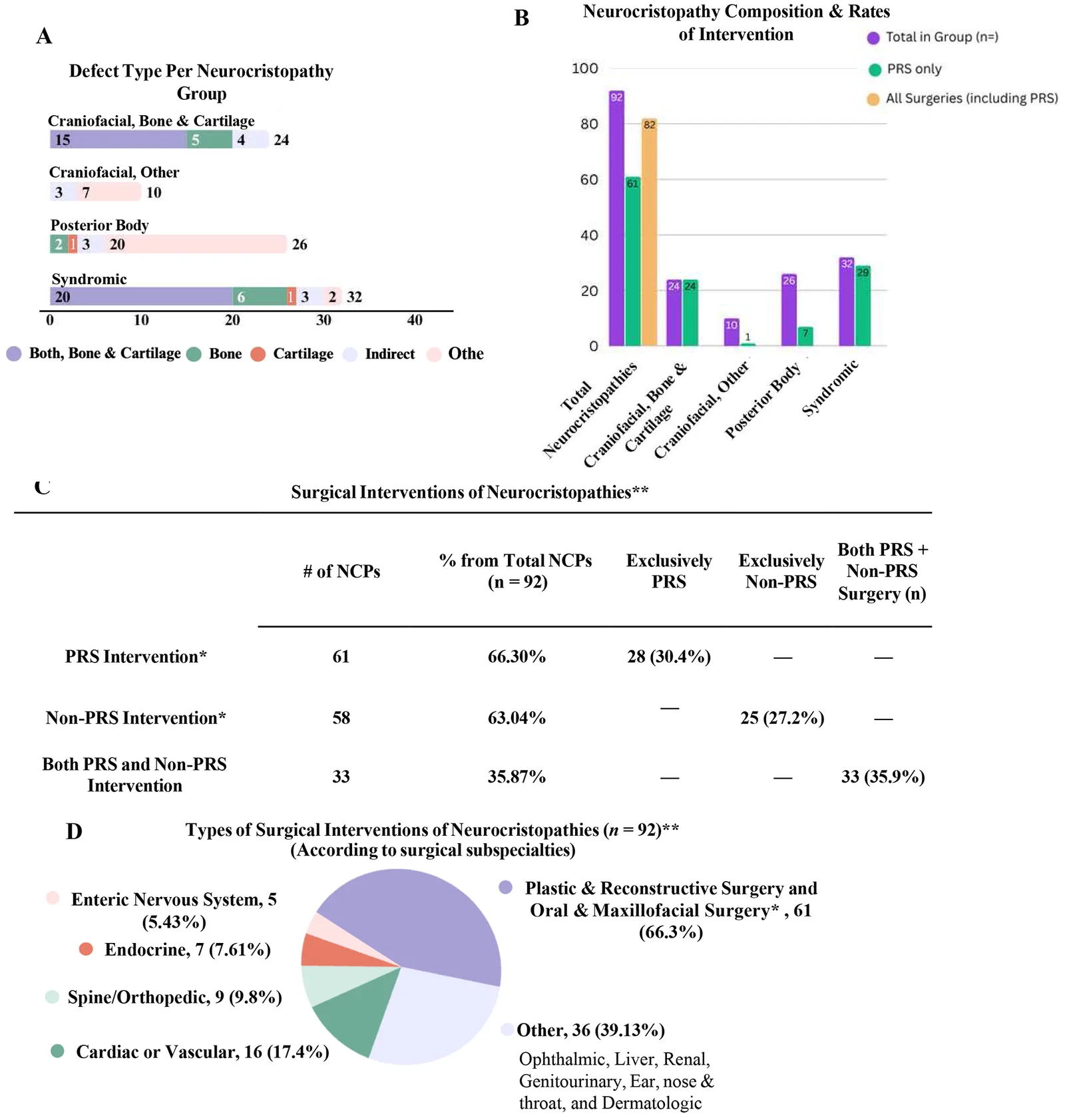
Defect type distribution and surgical intervention patterns across neurocristopathies. **(A)** Distribution of defect types across the neurocristopathy subgroups where defects were categorized as mixed bone and cartilage, bone-only, cartilage-only, indirect involvement, or “other”. The figure shows that the Syndromic group is the largest group of neurocristopathies with 32 diseases, which mainly consists of combined bone and carti lage as well as exclusive bone defects. Other defects are the second largest group (consisting of defects in various neural crest derived cell types, see Figure 3 and supplemental Data Table 1) with manifestation in 7 diseases in the “Cranofacial, other” and 20 diseases in the “Posterior Body” subgroups, respectively. **(B)** The graph demonstrates the proportion of neurocristopathies that require surgical intervention and specifically plastic and reconstructive surgery (PRS) out of all neurocristopathies (66%) and their respective subgroups. Statistical χ² analysis shows a significant difference in the requirement of PRS intervention in the craniofacial bone and cartilage group a s compared to both other craniofacial and posterior body subgroups (χ² = 51.7, df = 2, p < 0.0001). **(C)** A summary of surgical interventions across all NCPs, distinguishing between exclusively PRS/OMFS, exclusively non-PRS, and combined PRS plus non-PRS interventions. **(D)** Distribution of surgical subspecialties involved in NCP management highlighting PRS and major non-PRS categories. Note that surgical categories are not mutually exclusive, as individual NCPs may require interventions from multiple surgical specialties. *PRS and Oral & Maxillofacial Surgery (OMFS) have overlapping scope-of-practice in the craniofacial complex. Thus, PRS performed in the craniofacial region can be equally performed by OMFS and both specialties are grouped together in this category. ** Of note, neural crest-derived cancers (neuroblastoma, melanoma, pheochromocytoma, and paraganglioma), which all require surgical resection are n ot included in the statistics presented here.

The distribution of defect types varied significantly across the four NCP groups: Craniofacial, Bone & Cartilage (CFS B&C), Craniofacial, Other (CFS Other), Posterior Body, and Syndromic (encompassing defects in both the craniofacial and Posterior Body regions) (Figure 2C). Among the 24 CFS B&C cases, the majority (n = 15) exhibited mixed bone and cartilage defects, followed by bone-only (n = 5), and lastly indirect involvement (n = 4). In the CFS Other group (n = 10), most defects were categorized as “other”, indicating neither bone nor cartilage involvement (n = 7) and with fewer cases classified as indirect (n = 3) (Figure 4A). Similar to results for the CFS B&C group, most defects in the Syndromic group (n=32) were mixed bone and cartilage (n = 20), followed by bone-only (n = 6) and indirect (n = 3) (Figure 4A). The predominance of mixed bone and cartilage defects in the Syndromic group can be attributed to involvement of the craniofacial region and wide diversity of tissue types affected. In line with the body axis origin of NCC derived cell types (Figure 1 A–C), the Posterior Body group (vagal and trunk axial levels, n = 26) showed the highest proportion of cases with no bone or cartilage involvement (“other” n = 20), while indirect (n = 3), cartilage-only (n = 1), and bone-only (n = 2) defects were less common (Figure 4A). These findings highlight the predominance of structural skeletal defects, particularly mixed bone and cartilage involvement in craniofacial and syndromic NCPs, while NCPs in the posterior body more frequently exhibited indirect or non-skeletal defects such as congenital conotruncal malformation of the heart and intestinal innervation.

### 100% of craniofacial bone and cartilage anomalies require plastic surgery intervention

Among the 92 NCPs analyzed (Figure 3), 61 NCPs (66%) required plastic and reconstructive surgery (PRS). Involvement of Craniofacial symptoms significantly increased the likelihood of requiring PRS/OMFS, with 100% (24/24) of conditions in the Craniofacial, Bone and Cartilage (CFS B&C) group and 91% (29/32) of conditions in the Syndromic group requiring plastic surgery intervention. In comparison, 10% (1/10) of Craniofacial, Other conditions (CFS, Other) and 27% (7/26) of Posterior Body conditions required PRS (χ^2^ = 52.91, df = 3, p <0.001) (Figure 4B).

Defect type was a strong predictor of the need for plastic and reconstructive surgery (PRS). Mixed bone-and-cartilage defects showed the highest likelihood of requiring PRS, particularly within the Craniofacial Bone & Cartilage (CFS B&C) group (p < 0.001). Bone-only defects were also significantly associated with PRS intervention (p < 0.001). Relative risk analysis demonstrated that craniofacial neurocristopathies and syndromic neurocristopathies were 1.8 and 1.7 times more likely to require PRS than posterior body conditions respectively compared to other groups, underscoring the strong relationship between skeletal defects, craniofacial involvement, and reconstructive surgical need.

### Most NCPs require at least one surgical intervention, and many require multisystem management

While 61 NCPs required PRS, a significant subset, 58 NCPs (63%), required non-PRS surgical intervention (Figure 4C). Multisystem surgical involvement was common: 33 NCPs (35.9%) required both PRS and non-PRS procedures. Of the remainder, 28 NCPs underwent exclusively PRS, while 25 NCPs required exclusively non-PRS surgical management (Figure 4C). Given that 66.3% of NCPs require PRS and that NCPs collectively occur in 4.24% of live births, we estimate that approximately 2.81% of all live births are affected by an NCP requiring plastic and reconstructive surgery.

### Plastic surgery/Oral Surgery is the leading surgical specialty in NCP management, followed by diverse non-PRS subspecialties

PRS/OMFS was the most common surgical specialty involved in the management of neurocristopathies, with 61 of 92 NCPs requiring PRS/OMFS intervention (Figure 4D). Among non-PRS specialties, the largest category consisted of “Other” surgical services, including ophthalmology, ENT, dermatology, urology, renal, and hepatobiliary surgery, accounting for 36 NCPs (39.1%). This was followed by cardiac or vascular surgery in 16 NCPs (17.4%), spine/orthopedic procedures in 9 NCPs (9.8%), endocrine surgery in 7 NCPs (7.6%), and enteric nervous system (ENS) surgery in 5 NCPs (5.4%). These categories were not mutually exclusive; several NCPs required interventions across multiple surgical subspecialties (Figure 4D).

### Distribution of Functional, Aesthetic, and Mixed PRS Across Neurocristopathy Groups

**Figure 5:**
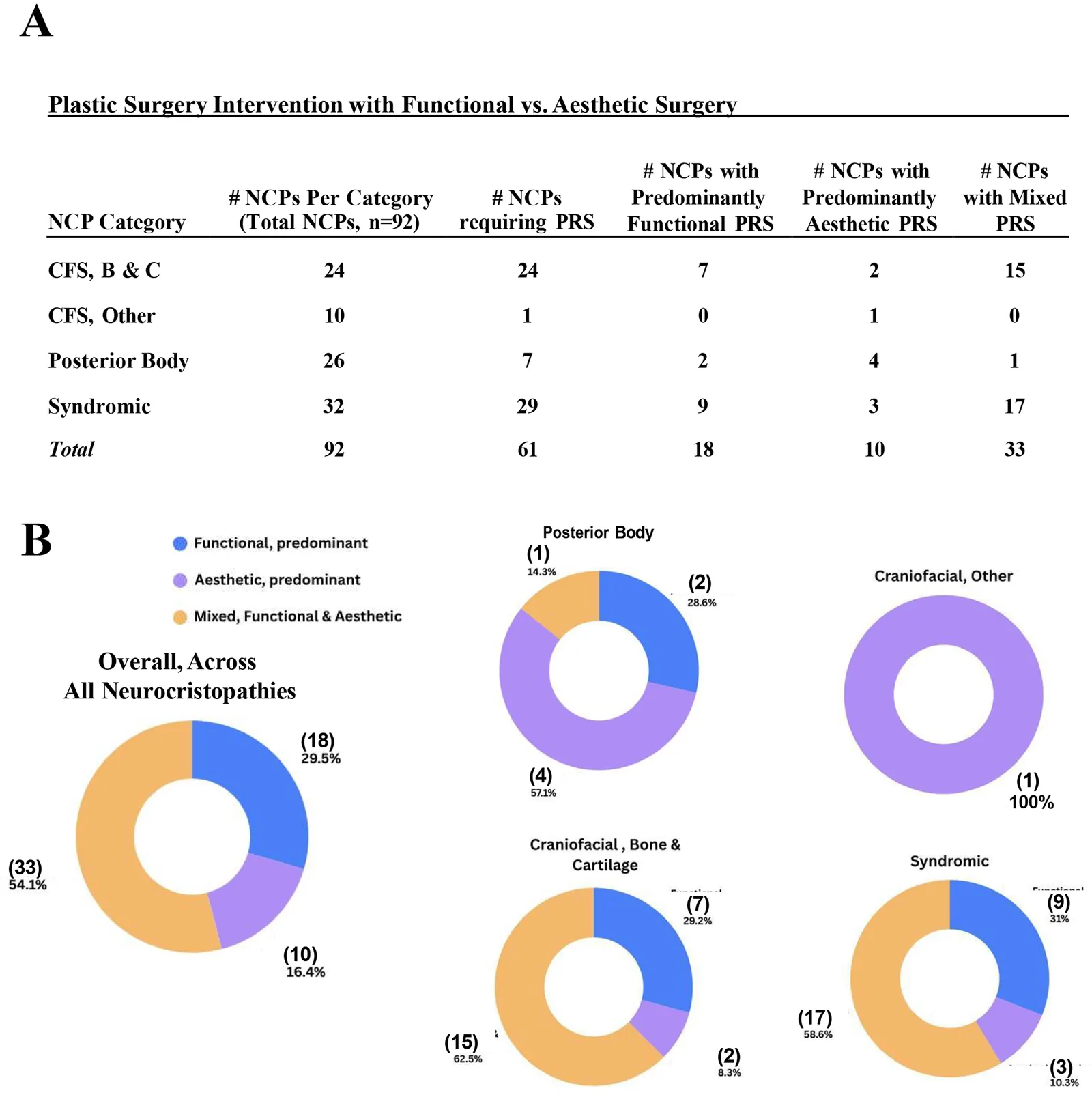
Functional versus aesthetic plastic surgery interventions across neurocristopathies. **(A)** A tabulation of plastic and reconstructive surgery (PRS) cases stratified by neurocristopathy (NCP) group and classification as predominantly functional, predominantly aesthetic, or mixed functional–aesthetic intervention, respectively as illustrated **(B)** by using donut plots, which show that, overall, mixed functional–aesthetic procedures was the dominant group in NCPs as well as in the Craniofacial Bone and Cartilage and Syndromic groups, whereas aesthetic procedures predominated the NCPs of Craniofacial, Other as well as the Posterior Body groups.

Across all neurocristopathies requiring plastic and reconstructive surgery (PRS), mixed functional–aesthetic procedures were most common, accounting for 54.1% (33/61) of cases, followed by functional-predominant operations in 29.5% (18/61) consisting of e.g. reconstructive surgery to improve breathing, feeding, speech, and vision. Finally, aesthetic-predominant surgeries like e.g. otoplasty and cleft lip repair remain vital for improving social outcomes, especially in cases of visible craniofacial anomalies that negatively affect self-image and confidence. These comprised 16.4% (10/61) of overall surgeries of NCPs (Figure 5A). In the Craniofacial Bone & Cartilage (CFS B&C) group, mixed procedures such as mandibular reconstruction and cleft repair were the dominating category (15/24; 62.5%), with functional (7/24; 29.2%) and aesthetic (2/24; 8.3%) interventions representing smaller proportions. The CFS Other group contained a single PRS case, which was aesthetic-predominant, whereas in the Posterior Body group, aesthetic-predominant procedures like removal of giant congenital nevus or melanosis were most frequent (4/7; 57.1%), followed by functional (2/7; 28.6%) and mixed (1/7; 14.3%) interventions (Figure 5B). Similar to the trends seen for the CFS B& C group, the Syndromic group had a predominance of mixed procedures due to the involvement of the craniofacial region (17/29; 58.6%) followed by functional PRS procedures (9/29; 31.0%) and aesthetic (3/29; 10.3%) interventions representing smaller proportions (Figure 4A, B). The overall distribution of functional, aesthetic, and mixed PRS procedures differed significantly between anatomical groups (χ^2^ = 16.29, df = 6, p = 0.0123), indicating that the type of reconstructive need is influenced by the underlying neurocristopathy location.

### Genes linked to peripheral nervous system development are frequently associated with neurocristopathies

When genetic analysis was addressed, information from our set of references revealed that several transcription factors and signaling molecules essential for neural crest cell (NCC) development are recurrently implicated across neurocristopathies (NCPs). *PHOX2B* was a frequently associated gene, appearing in context of 8 NCPs, consistent with its critical role in autonomic and peripheral nervous system development (22–36). Next, *SOX10*, a master regulator of early neural crest specification as well as later differentiation into glial cells, Schwann cell precursors, and melanocytes, was associated with 7 NCPs (6, 8, 22, 37–51). Additionally, *RET*, the receptor for GDNF-family ligands required for development of NCC-derived sensory neurons and enteric ganglia, was implicated in 6 NCPs (21, 31, 52–62). Additional genes including *TP63, EDNRB, PAX3, GDNF, TWIST1, FGFR2,* and *CHD7* were each associated with three or more neurocristopathies, underscoring their broad roles in NCC migration, differentiation, and craniofacial patterning (6, 29, 39, 43, 63–70).

When examining conditions rather than individual genes, Hirschsprung disease exhibited the highest number of known pathogenic mutations (26 total variants) (21, 24, 29–36, 39, 43, 48, 50, 61, 71–76), followed by craniosynostosis syndromes (13 variants) (1–3, 5, 15, 76–79), many of which involve *FGFR2* and *TWIST1* mutations affecting NCC-derived craniofacial bone. 22q11.2 deletion syndrome was linked to 11 NCPs (21, 34, 80–87), reflecting the multisystem impact of deletions affecting *TBX1* and other NCC-related genes (80, 82–84). Collectively, these findings highlight the genetic diversity underlying NCPs and the central contribution of NCC-associated pathways to their pathogenesis. Of note, because addressing the genetic mutations involved in a given neurocristopathy was not a requirement for inclusion among the references used in this study, the proportional prevalence of the genetic alterations reported here should not be interpreted as statistically meaningful.

### Majority of plastic surgery interventions of NCPs consist of mandibular, cleft lip and palate, and micrognathia repair

**Figure 6:**
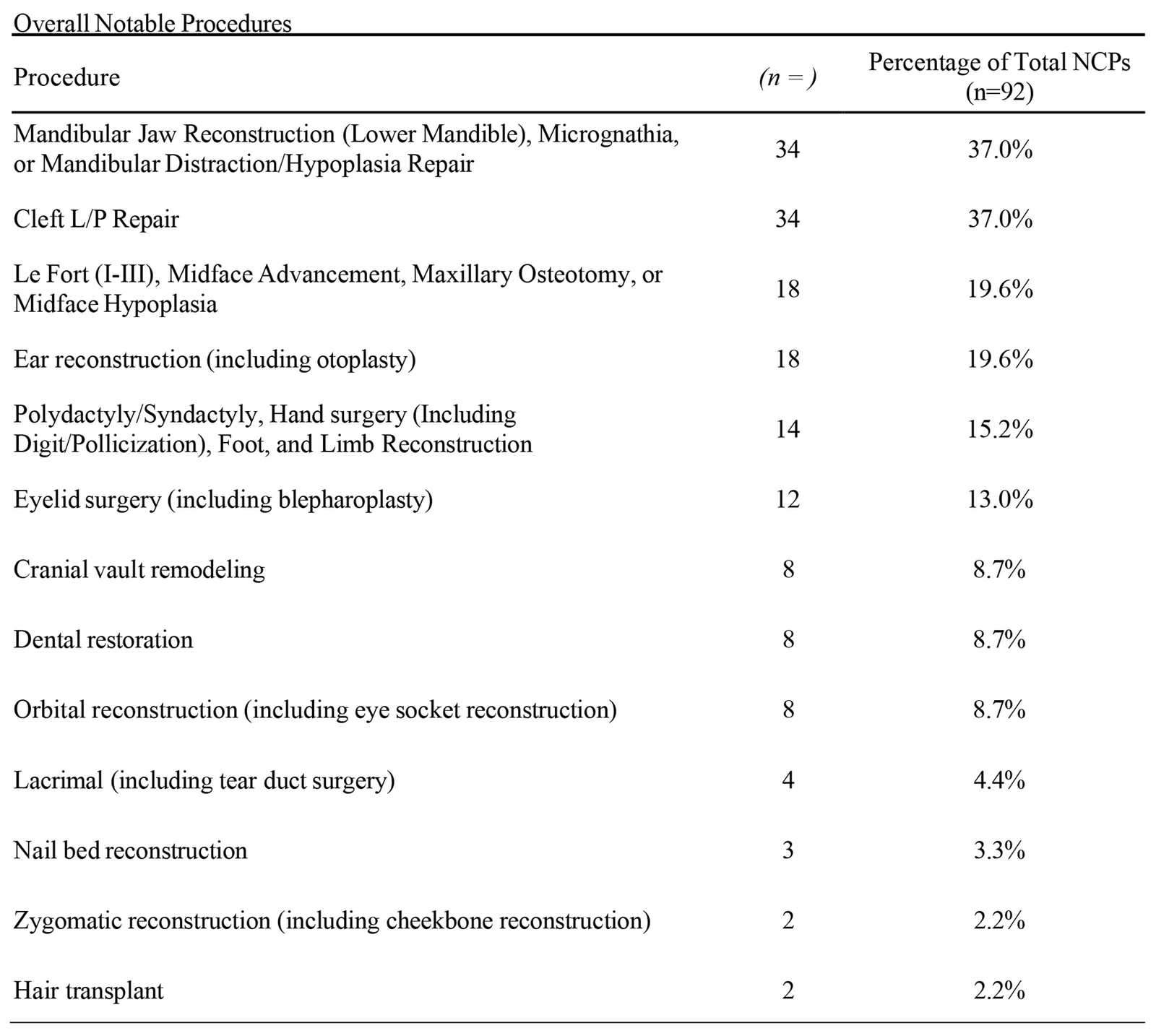
Distribution of notable plastic and reconstructive procedures across neurocristopathies. Tabulation summarizes the most frequently reported plastic and reconstructive surgery (PRS) procedures associated with neurocristopathies (NCPs) (n = 92). Mandibular reconstruction (including micrognathia correction and mandibular distraction or hypoplasia repair) and cleft lip and/or palate repair were the most common interventions, followed by midface advancement procedures (Le Fort I–III), ear reconstruction, and limb reconstruction. Percentages reflect the proportion of total NCPs associated with each procedure. Note that the procedures are not mutually exclusive, as individual NCPs may require multiple surgical interventions.

To further characterize the surgical needs of neurocristopathies (NCPs), we analyzed the specific reconstructive procedures performed across conditions. Mandibular reconstruction, including lower mandibular advancement, distraction osteogenesis, micrognathia correction, and hypoplasia repair, was among the most frequently performed intervention, occurring in 34 NCPs (37.0%). This reflects the high prevalence of mandibular skeletal deficiency across craniofacial NCPs and the critical functional importance of the mandible for airway stability, feeding, occlusion, and facial symmetry. Similarly, cleft lip and/or palate repair was also the most common intervention, performed in 34 NCPs (37.0%), underscoring the strong association between NCC dysfunction and orofacial clefting (Figure 6).

Procedures addressing midface dysmorphology, including Le Fort I–III osteotomies and midface advancement, were performed in 18 NCPs (19.6%), while ear reconstruction (including otoplasty and microtia repair) alson occurred in 18 NCPs (19.6%). Beyond the craniofacial region, polydactyly/syndactyly and other limb reconstructions were required in 14 NCPs (15.2%), reflecting the syndromic nature of several NCPs in which non-NCC derived limb anomalies occur alongside NCC-specific defects. Although these limb anomalies are not NCC-derived, they co-occur in disorders involving shared developmental pathways, such as ciliopathies and Rasopathies (5, 88–92). In these conditions, disruption of signaling pathways (e.g., Hedgehog, Wnt, and Notch) that are essential for both neural crest and limb development can result in indirect skeletal anomalies (47, 63, 84, 91, 93–102).

Additional procedures included eyelid surgery (13.0%), cranial vault remodeling (8.7%), dental restoration (8.7%), orbital reconstruction (8.7%), lacrimal duct surgery (4.4%), nail-bed reconstruction (non-NCC derived) (3.3%), and zygomatic or cheekbone reconstruction (2.2%). Collectively, these findings highlight the breadth of reconstructive demands in NCPs and reinforce the importance of comprehensive, multisystem surgical planning in conditions where craniofacial, neurologic, and systemic anomalies intersect.

## Discussion

This study provides a comprehensive analysis of the prevalence, anatomical implications, and surgical needs of NCPs, emphasizing the dual focus of PRS in restoring functional and aesthetic outcomes. Furthermore, our findings underscore the critical role of surgery, especially plastic and reconstructive surgery in managing neurocristopathies, particularly those involving craniofacial anomalies.

### Prevalence of NCPs is substantially higher than previously thought

The cumulative prevalence analysis estimated NCPs at 4.24% of live births. With global data showing 6–7% of live births result in congenital anomalies, our findings suggest NCPs may account for up to 60–70% of these defects and uncovers a significant underestimation of regularly used claims that 15–30% of birth defects are NCP-related (3, 16–18, 74, 79, 103–105). This study provides the first comprehensive quantification of NCP prevalence using objective data, questioning long-standing claims while advocating for more precise documentation and recognition of NCP-related birth defects and their severity (Figure 2). Although we invested systematic effort to cover all NCPs, the list of 92 diseases is likely to still modestly increase in the future. Similarly, as the total number of birth defects may also be an underestimation due to overall incomplete reporting of disease prevalences, the calculated 60-70% should be taken with caution, and we suggest using over 50% instead. Furthermore, the percentage of NCPs of all birth defects may be even higher as our results indicate that in addition to the 64% of NCPs requiring plastic surgery intervention, ~35% of NCPs consist of milder anomalies, but precise estimates are challenging to calculate as there is no accurate estimate of the percentage of all birth defects that include also mild cases.

### Surgical Diversity Reflects Multisystem Involvement in NCPs:

The surgical distribution across neurocristopathies (NCPs) reflects their inherently multisystem nature. Nearly two-thirds of NCPs in the analysis required plastic and reconstructive surgery (PRS), emphasizing the substantial reconstructive demands imposed by these congenital anomalies (Figures 4–5). While PRS/OMFS represented the most frequently involved surgical specialties, contributions from non-PRS fields were also notable and anatomically diverse (including cardiac, enteric nervous system, endocrine, and other surgeries). Visualization of surgical types illustrates that, although PRS involvement spans all NCP categories, non-PRS procedures were more variable and typically aligned with the underlying organ systems affected in each condition. This pattern reflects the wide range of neural crest - derived cell types throughout the body and underscores the heterogeneous presentation of NCPs, while reinforcing that, despite broad anatomical involvement, craniofacial anomalies consistently require specialized PRS care. These findings emphasize the need for multidisciplinary coordination in managing NCPs, with PRS playing a central but not exclusive role in addressing their complex surgical needs.

### Craniofacial Involvement and Surgical Demand

Syndromic and craniofacial neurocristopathies (NCPs), particularly those affecting bone and cartilage in the craniofacial region(CFS B&C, Syndromic), exhibited the highest surgical burden among all anatomical groups (Figure 4), consistent with the fact that only cranial NCCs differentiate into bone and cartilage (Figure 1). All conditions in the CFS B&C category (n = 24) required PRS/OMFS, reinforcing its central role in managing craniofacial skeletal anomalies. While craniofacial conditions categorized as “Other” (CFS Other, n = 10) showed a more variable pattern of involvement, the majority of cases in the combined craniofacial group still required PRS/OMFS intervention. In contrast, the Posterior Body group (n = 26), which includes cardiac, enteric nervous system, thyroid, thymus, and other anomalies, exhibited a lower rate of PRS need and a greater proportion of non-PRS procedures. These patterns highlight the anatomically driven surgical variability of NCPs and underscore the disproportionate reconstructive burden imposed by craniofacial anomalies, necessitating targeted surgical expertise and multidisciplinary care.

### Defect Type as a Predictor of Surgical Complexity

The type of structural defect emerged as a key predictor of PRS involvement across NCPs. Mixed bone and cartilage defects were most frequently associated with surgical intervention, followed by bone-only anomalies. In the craniofacial group, particularly CFS B&C, mixed and osseous defects accounted for nearly all cases, reinforcing their high reconstructive complexity. Relative risk analysis confirmed that craniofacial NCPs were over twice as likely (RR = 2.25) to require PRS/OMFS compared to non-craniofacial counterparts. These findings emphasize the clinical value of early defect-type identification in surgical planning and resource allocation. Improved recognition of skeletal involvement, especially in early diagnosis, can guide timely multidisciplinary referrals, optimize intervention timing, and ultimately improve functional and aesthetic outcomes for patients with NCPs.

### Functional and Aesthetic Surgical Needs in NCPs

Mixed functional & aesthetic surgeries predominated across all NCPs, particularly in craniofacial bone & cartilage cases and Syndromic cases with craniofacial involvement, reflecting the need to address physiological impairments such as feeding, breathing, and speech. Aesthetic procedures, while less frequent, remain vital for improving social outcomes, especially in cases of visible craniofacial anomalies that hinder social integration by negatively affecting self-image, confidence, and overall quality of life (106–108). Specific procedures, such as mandibular reconstruction, cleft lip/palate repair, and midface advancement, illustrate this dual impact. Mandibular reconstruction, tied with cleft L/P repair for the most frequently performed procedure, restores jaw function and simultaneously improves facial appearance in mandibular hypoplasia, while cleft repair addresses significant impairments in orofacial function. Midface advancements enhance breathing, vision, and facial symmetry, demonstrating the intersection of functional recovery and aesthetic refinement in craniofacial reconstruction. Together, these procedures highlight the essential balance in PRS between restoring function and addressing aesthetic concerns to meet the comprehensive medical and psychosocial needs of patients with NCPs.

### Genetic associations in NCP

The high frequency of *PHOX2B*, *SOX10*, and *RET* mutations in NCPs reflect their critical role in neural crest development and further differentiation of autonomic nervous system, pigmentation, and craniofacial structures. Hirschsprung disease, with 26 mutations (21–24, 29–36, 39, 43, 48, 50, 61, 71–76, 90, 109–121), reflects the complexity of enteric nervous system formation and genetic susceptibility to malformations, while craniosynostosis involves multiple genes regulating skull suture patency and closure. Identifying these genetic hotspots provides insight into screening, precision medicine, and early intervention strategies for NCPs, emphasizing the need for further research on gene-environment interactions and their clinical implications.

### Guideline Recommendations

While current guidelines from the American Society of Plastic Surgeons (**ASPS**) (122), American Cleft Palate–Craniofacial Association (**ACPA**) (123), and American Association of Oral and Maxillofacial Surgeons (**AAOMS**) (124) provide standardized timelines for common procedures such as cleft lip/palate repair and cranial vault reconstruction, they primarily focus on isolated craniofacial anomalies. These frameworks often fail to address the complexity of syndromic conditions involving the craniofacial complex, particularly those classified as NCPs with overlapping cardiac, enteric, neurologic, and pigmentary manifestations (79, 104, 105, 122–125). The absence of defect-specific pathways for rare and multisystem NCPs limits their utility in clinical decision-making.

Our findings highlight the need for early functional interventions to address impairments in feeding, breathing, and speech. As discussed earlier, visible craniofacial anomalies are also associated with reduced self-esteem, social withdrawal, and impaired quality of life, underscoring the importance of incorporating psychosocial goals into surgical planning. Aesthetic procedures, while less frequent, play a vital role in addressing these challenges and are often necessary in staged reconstruction to support longterm psychosocial well-being (106–108).

Additionally, the high frequency of procedures such as mandibular reconstruction, cleft lip/palate repair, and midface advancement within NCPs reinforces the need for refined, targeted guidance. These insights suggest several opportunities to enhance existing guidelines:

1. Defect-Specific Recommendations: Develop tailored timelines for both functional and staged aesthetic procedures based on defect type, anatomical region, and syndromic context, bridging current gaps in ASPS, AAOMS, and ACPA frameworks.
2. Integrated Functional and Aesthetic Goals: Ensure that surgical planning addresses both physiological impairments and psychosocial outcomes, especially in cases of visible craniofacial anomalies that impact self-image and social development.
3. Integrated surgical planning of syndromic NCPs that comprise of defects in multiple tissues across the body: Ensure that surgical planning addresses all future procedures across surgical subspecialties to tailor timelines that reduce surgical burden and optimize patient outcome and recovery time.
4. Improved Documentation of Rare NCPs: Standardize classification and reporting practices for underrepresented NCPs to strengthen prevalence data and guide future evidence-based recommendations.
5. Expanded Multidisciplinary Team Approaches: Strengthen collaboration across plastic surgeons, oral surgeons, dentists, orthodontists, speech-language pathologists, geneticists, pediatricians, and developmental biologists. Incorporating insights from both clinical and basic science perspectives is essential to improve care coordination and to elucidate the cellular and molecular mechanisms driving complex NCP phenotypes.

By building on current ASPS, AAOMS, and ACPA frameworks, these recommendations aim to provide a more inclusive, evidence-based approach to managing NCPs. Incorporating these refinements into existing guidelines could guide surgeons in optimizing interventions while addressing the unique challenges posed by NCPs, ultimately improving patient outcomes and quality of life.

## Conclusions

This study underscores the critical role of plastic and reconstructive surgery in managing neurocristopathies, particularly in craniofacial anomalies that involve mixed bone and cartilage defects, which exhibited the highest demand for surgical intervention. We demonstrate that functional surgeries predominate the group of neurocristopathies that required plastic and reconstructive surgery, addressing critical impairments in feeding, breathing, and speech, while aesthetic procedures remain essential for enhancing psychosocial well-being and social integration.

By analyzing NCP prevalence and surgical needs, this work challenges historical estimates, providing evidence that NCPs account for a greater proportion of congenital anomalies than previously recognized, up to 60-70% of all birth defects in live births. Insights into procedure-specific trends, including the prevalence of cleft lip/palate repair and mandibular reconstruction, emphasize the importance of defect-specific surgical planning and resource allocation.

These findings highlight the need to refine clinical guidelines by incorporating tailored timelines for functional and aesthetic surgeries, improving documentation of rare conditions, and fostering interdisciplinary collaboration among a multidisciplinary team. Such updates can better guide surgical timing and enhance outcomes for complex craniofacial anomalies.

Ultimately, this research affirms the critical role of plastic and reconstructive surgery in restoring function, improving quality of life, and addressing the multifaceted challenges of NCPs. By advancing understanding of NCP etiology, epidemiology and surgical implications, this study provides a foundation for innovation in reconstructive surgery and the development of targeted, evidence-based strategies to improve long-term patient outcomes (126, 127).

## Methods

A scoping review of the literature was performed adhering to PRISMA (Preferred Reporting Items for Systematic Reviews and Meta-Analyses) 2020 guidelines was conducted to compile a comprehensive list of NCPs and analyze the relationship between craniofacial involvement and the need for PRS. Searches were performed in PubMed and cross-referencing of MEDLINE and Scopus using the following terms (and their combinations): [“neurocristopathies” OR “neurocristopathy] AND [“abnormalities” OR “anomalies” OR “birth” AND “defects“ OR “birth defects” OR “congenital abnormalities” OR “congenital” AND “abnormalities” OR “congenital anomalies”]. All search results were manually screened by two independent screeners using Covidence software. Suitability for inclusion was determined upon title and abstract screening and again following full text review with any conflicts between independent screeners discussed and resolved before final analysis. The search strategy ensured a systematic and reproducible approach to identify relevant literature based on which a cumulative table summarizing NCPs and their prevalence, embryological origins, and specific details was created. Additionally, publications that were referenced by the original hits were added to complement the literature analysis (Figure 2A).

### Neurocristopathy Group Categorization

Identified NCPs were categorized into four main groups (Figure 3): **(1)** Craniofacial, Bone and Cartilage (**CFS B&C**), which consists of conditions primarily affecting craniofacial bone and cartilage. **(2)** Craniofacial, Other (**CFS Other**), encompassing conditions that impact functions of NCC-derived craniofacial structures (e.g., vision, hearing, olfaction) that are derived from impaired neural crest-derived tissue, like compromised crosstalk of the (NCC-derived) peripheral nervous system with the sensory placodes. In other words, diseases in this category are primary NCPs but without direct developmental impact on bone and cartilage. **(3)** Posterior body, covering conditions involving non-craniofacial structures with confirmed NCC contributions at the vagal and trunk body axes, such as the enteric nervous system (e.g., Hirschsprung disease), cardiac outflow tract (e.g., conotruncal anomalies), thymus,thyroid, parathyroid and adrenal medulla. **(4)** Syndromic, including conditions that involve structures in both the craniofacial as well as the Posterior body regions (Figure 1).

### Type of Surgical Interventions

To evaluate surgical intervention patterns, we categorized procedures associated with each NCP into three primary groups (Figure 4C–D): (1) Plastic and Reconstructive Surgery (PRS) intervention, (2) non-PRS surgical intervention, and (3) cases involving both PRS and non-PRS interventions. Non-PRS surgical interventions were further subclassified by anatomical system, including cardiac or vascular, endocrine, enteric nervous system (ENS), spine/orthopedic, and other specialties (e.g., Ophthalmic, hepatic, renal, genitourinary, ear nose, and throat (ENT), and dermatologic). In the Craniofacial region, there is significant overlap in scope-of-practice for PRS and Oral & Maxillofacial Surgery (OMFS) and both groups contribute equally to surgeries in this region. Since most of the NCPs were found to comprise of Craniofacial surgeries, we provide insight for both PRS and OMFS. Of note, neural crest-derived cancers (neuroblastoma, melanoma, pheochromocytoma, and paragangliomas), which all require surgical resection are not included in the statistics presented here. The frequency and percentage of each intervention type were calculated relative to the total number of NCPs (n = 92), with particular attention to patterns of overlap across surgical specialties. Visual representations of these distributions were created using R and the ggplot2 package, including bar plots and pie charts to highlight the relative contributions of PRS and other surgical subspecialties to the overall surgical burden of NCPs. Importantly, although some of these organ systems are not neural crest–derived, they require treatment in NCPs because secondary effects of craniofacial or systemic malformations can impact their structure or function. For example, in Alagille syndrome, patients may require liver transplantation or corrective cardiac surgery in addition to craniofacial procedures such as eyelid ptosis repair or mandibular reconstruction. Similarly, Bardet–Biedl syndrome often necessitates functional surgeries such as polydactyly correction, bariatric procedures, or renal transplantation, with PRS involvement for ear reconstruction or facial contouring. In Mowat–Wilson syndrome, surgical care spans both functional and aesthetic domains, including Hirschsprung disease repair, genitourinary reconstruction for hypospadias, craniofacial reconstruction, and scar revision. Together, these examples underscore the multisystem and multidisciplinary surgical needs of NCPs.

### Type of Bone and Cartilage Defect Classification

Defects associated with NCPs were classified into five categories: cartilage-only, bone-only, mixed bone and cartilage, indirect involvement, and other. Cartilage- and bone-only defects were defined as those predominantly involving either cartilage- or bone-only. Mixed defects included both cartilage and bone. “Indirect involvement” was used to describe defects primarily affecting other structures but with secondary involvement of bone or cartilage such as Cochleovestibular Nerve Malformations. “Other” indicated no bone or cartilage involvement. This classification enables a focused assessment of reconstructive requirements across the different primary defect types, providing insight into the complexity of surgical intervention specific to each condition.

### Functional vs. Aesthetic Plastic Surgery Involvement

Additionally, we also analyzed the nature of PRS involvement by distinguishing between functional and aesthetic interventions. Functional surgeries include those aimed at restoring essential physiological functions compromised by structural abnormalities, such as breathing, feeding, speech, and sensory abilities. In contrast, aesthetic surgeries focus on enhancing physical appearance and addressing both cosmetic and psychosocial aspects of patient care, particularly in cases of facial anomalies. For the purposes of this analysis, commonly performed aesthetic procedures such as scar revision, skin grafting, and facial contouring were excluded from the count, as these interventions are frequently used across a wide range of conditions and are not exclusive or specific to the congenital structural abnormalities seen in NCPs. Including them would have introduced bias by overrepresentation of aesthetic interventions that are often adjunctive or secondary in nature, rather than central to the core pathology of NCPs.

### Notable Plastic Surgical Procedures in Neurocristopathies

Data was collected on the most common PRS procedures associated with NCPs. These procedures were identified based on frequency across documented cases and included both craniofacial and non-craniofacial interventions. While many of these procedures involved craniofacial reconstruction including cleft lip and palate repair, mandibular reconstruction, and LeFort osteotomies, others, including hand and limb reconstructions that were part of multi- syndromic anomalies also affecting tissues not derived from neural crest, were also represented.

### Cumulative Incidence and Prevalence of NCPs

To evaluate the un-documented claims that 15–30% of birth defects consist of NCPs (3, 16–18), we collected reported prevalence data for individual NCPs in a thorough review process. Prevalence rates were sourced from published studies, standardized to a per-thousand metric, and cumulatively aggregated. For NCPs with extremely low prevalence (fewer than 100 documented cases worldwide), a conservative estimate of one in a million was applied to ensure inclusion in the cumulative analysis while avoiding inflation of overall prevalence. This threshold was chosen to maintain methodological consistency and minimize bias, as only a few NCPs were reported to occur less frequently than this rate (e.g., 1 in 10 million). Although there is no universally standardized prevalence cut-off for ultra-rare diseases, our approach aligns with rare disease epidemiology practices, which often adapt estimations based on available data and the need to prevent overrepresentation of minimally reported conditions (128). This strategy also reflects findings from analyses of databases like Orphanet, where the majority of rare diseases exhibit a point prevalence below 1 in a million (129). This data-driven approach aimed to provide an accurate estimation of NCP prevalence and correct historically used non-evidence-based prevalence estimates used in the field. Cumulative incidence was contextualized against the global population of 7.9 million annual births affected by serious congenital anomalies (6% of all births) (11, 15, 130), highlighting the proportion of birth defects attributable to NCPs.

### Statistical Analysis

To evaluate the relationship between anatomical group, defect type, and the likelihood of requiring PRS, chi-square tests were used to compare intervention rates among the different NCP groups. A p-value of <0.05 was considered statistically significant. To quantify the association between craniofacial involvement and the likelihood of PRS, relative risk (RR) calculations were performed. Comparisons were made between CFS and non-CFS cases, with sub-analyses for defect types (e.g., mixed bone and cartilage, bone-only, and cartilage-only). Functional and aesthetic surgeries were analyzed separately to determine their prevalence across groups. Proportional differences in intervention rates for functional and aesthetic procedures were assessed using chi-square tests to identify trends and statistical significance. Descriptive statistics were used to report the frequencies of specific surgical procedures.

### Tools and Software

All statistical analyses were conducted using Microsoft Excel (Version 2504) and RStudio (Version 2025.05.0), and results were summarized in tables and charts to depict surgical intervention rates, defect classifications, and procedure frequencies. Figures, schematics, and visual illustrations were generated or formatted using BioRender.com, Canva, and RStudio. Covidence software was used for article screening, filtering, and full text review to determine suitability for inclusion in review.

## Supplementary Material

1

## Data Availability

All data produced in the present work are contained in the manuscript
