# Supplementary material for: Unveiling the Prevalence and Surgical Burden of Neurocristopathies: A Scoping Review and Statistical Analysis
Source: Res Sq. 2026 Aug 6:rs.3.rs-10318477. Preprint. [Version 1] doi: 10.21203/rs.3.rs-10318477/v1 (PMC13484851; doi:10.21203/rs.3.rs-10318477/v1)
Supplement: Supplementary file 1 [file NIHPPrs10318477v1-supplement-1.pdf]

Supplemental Table 2

**Neurocristopathy (NCP)**	References
22q11.2 Deletion Syndrome/Di George anomaly	(3, 21, 34, 69, 80-87, 131)
3MC Syndrome / Malpuech-Michels-Mingarelli-Carnevale syndrome	(132-134)
[The] 6p25 deletion syndrome/Axenfeld-Rieger Syndrome	(135-138)
ADULT (Acro-Dermato-Ungual-Lacrimal-Tooth Syndrome) Syndrome	(63, 139)
AEC Syndrome/Ankyloblepharon-Ectodermal defects-Clefting/ Hay-Wells syndrome	(63, 140, 141)
Alagille Syndrome	(113, 142-144)
Allgrove Syndrome/Triple-A syndrome (achalasia-addisonianism-alacrima syndrome)	(145-148)
Aortic Aneurysm / Arches	(16, 126, 127)
Auriculo-Condylar Syndrome	(65, 149-152)
Aplasia Cutis Congenita (ACC)	(153)
Bamforth-Lazarus Syndrome	(154, 155)
Bardet -Biedl Syndrome 8	(156, 157)
Binder Syndrome/ maxillonasal dysplasia or nasomaxillary hypoplasia<br>Binder's Syndrome with Dysautonomia	(158-160)
Branchio-Oculo-Facial Syn.	(161-163)
BOR (Branchio-Oto-Renal) Syndrome/Melnick-Fraser syndrome	(164-168)
Charcot-Marie-Tooth	(40, 169, 170)
Craniosynostosis	(1-3, 5, 78, 79, 171)
CHARGE Syndrome (Coloboma (eye defects), Heart defects, Atresia of the choanae (blockage of the nasal passages), Retarded growth and development	(67, 69, 70)
Orofacial Cleft<br>Orofacial cleft 5 (OFC5, MSX1 predominance)	(61, 172-174)
Cochleovestibular Nerve Malformations	(168, 175)
Congenital Central Hypoventilation Syndrome (Ondine's Curse)	(21, 24, 31, 52-55)
Congenital Pupillary-Iris-Lens Membrane with Goniodysgenesis	(53, 117, 176, 177)
Cranioectodermal dysplasia/Sensenbrenner syndrome	(77, 89, 91, 92, 178)
Craniofacial-Deafness-Hand Syndrome	(39, 47, 179)
Craniofacial anomalies associated with Kearns-Sayre Syndrome	(22-25, 180)
CSC-KT (Cardiofaciocutaneous syndrome, Keratosis Follicularis Spinulosa Decalvans (KFSD), and Tangier disease)	(5, 181)
Diamond Blackfan Anemia Syn.	(100, 182)
EEC3 Syndrome	(63, 101, 183, 184)
Ellis-van Creveld syndrome	(91, 185-187)
Facial anomalies in cytochrome-oxidase deficiency (and subsequent Kearns-Sayre syndrome with growth hormone deficiency)	(180)
Frontonasal Dysplasia/median cleft face syndrome	(115, 188, 189)
Familial dysautonomia/Riley-Day syndrome	(190-192)
Fryns Syndrome with Hirschprung Disease	(193, 194)
Fusiform Aneurysm	(195)
Giant Congenital Melanocytic Nevus	(196-199)
Goldberg–Shprintzen Megacolon Syndrome (GSMS)	(29, 72)
Goldenhar's syndrome/Oculo-Auriculo-Vertebral Syndrome	(21, 200)
Gorlin-Goltz Syndrome/basal cell carcinoma syndrome	(201, 202)
Growth Retardation, Developmental Delay, And Facial Dysmorphism	(203, 204)

Hajdu-Cheney Syndrome	(111, 112, 114)
Haddad syndrome (Ondine-Hirschsprung syndrome)	(31, 205, 206)
Hirschsprung	(21, 24, 29-36, 39, 43, 48, 50, 61, 71-76)
Hypogonadotropic Hypogonadism/Kallmann Syndrome	(46, 48-51, 68, 78, 207)
Hypohidrotic Ectodermal Dysplasia	(63, 96, 208-210)
Johnson-McMillin Syndrome	(211)
JS-X syndrome	(212)
Kabuki Syndrome	(5, 119, 213-215)
Klippel-Feil Syndrome	(66, 216-220)
Lattice corneal dystrophy	(221-224)
Leigh syndrome	(225-227)
Limb-Mammary	(63, 101, 102)
Lujan-Fryns Syndrome	(228-232)
Meckel-Gruber Syndrome	(90, 93, 233-235)
Medullary Thyroid Carcinomas	(56-60, 62)
MED13L Haploinsufficiency Syndrome (MRFACD / Asadollahi-Rauch Syndrome)	(236-238)
Melanoma	(239-242)
Mandibulofacial Dysostosis with Alopecia	(243, 244)
Median Cephalic Neurocristopathy	(245)
Moebius Syndrome	(174, 246-249)
Mowat Wilson Syndrome	(34, 109, 250)
Moyamoya Disease	(4, 251-253)
Multiple Endocrine Neoplasia Type 2 (MEN2)	(1, 57, 58, 60, 83, 254-256)
Multiple Sclerosis	(26)
Musculocontractural Ehlers-Danlos syndrome (mcEDS)	(120, 257, 258)
Neurocutaneous Syndromes	(7, 47, 259, 260)
Neurocutaneous melanosis	(197-199)
Neuroblastoma	(23, 24, 27, 28)
Noonan syndrome	(86, 88, 116, 213, 261-264)
Oculocutaneous Albinism	(265, 266)
Oculocerebrocutaneous (Delleman) Syndrome	(267, 268)
Orofaciodigital Syndrome I	(90, 269)
Paragangliomas	(110, 270, 271)
PCWH, Waardenburg predominance (Peripheral demyelinating neuropathy, central dysmyelinating leukodystrophy, Waardenburg syndrome and Hirschsprung disease)	(21, 37, 39, 41, 43-45, 272)
Peter's Anomaly	(273-277)
PHACE (Posterior fossa malformations, Hemangiomas (large vascular birthmarks on the face), Arterial anomalies, Cardiac defects, Eye abnormalities, and Sternal Breast Bone Defects)	(4, 245, 259, 260, 278)
Pheochromocytomas	(21, 60, 62, 270, 271, 279-281)
Piebaldism	(282, 283)
Pierre Robin sequence	(112, 284-289)
Postaxial Acrofacial Dysostosis Syndrome (Miller Syndrome)	(186, 290)
SAMS (Short Stature, Auditory Canal Atresia, Mandibular Hypoplasia, and Skeletal Abnormalities) Syndrome	(256, 291, 292)
Seckel Syndrome / Microcephalic Osteodyplastic Primordial Dwarfism, Type II (MOPD2)	(293-296)
Sensorineural Hearing Impairment	(121)
Short-Rib Thoracic Dysplasia	(89, 91, 92, 297)
Split-hand/split-foot malformation 4	(63, 64, 98, 99, 101)
SUPT16H-associated neurodevelopmental disorder and neurocristopathy	(298)
Tietz Syndrome	(118, 299)
Treacher Collins Syndrome	(6, 125, 300-303)
Tricho-dento-osseous Syndrome	(304-307)
Van der Woude Syndrome	(308)

Vascular Malformations (Hypoplastic Internal Carotid Artery)	(4, 69, 74, 84, 204, 251, 252, 309)
Von Hippel-Lindau Syndrome	(256, 281, 310-312)
Yemenite deaf-blind hypopigmentation syndrome	(42, 313)

Neurocristopathy (NCP)	NCP Category: Craniofacial (Bone & Cartilage, CFS), CFS, Other (CO, i.e. sensory function, eyes, optic nerves, ear structures), Posterior Trunk (PT), Syndromic (SYN)	Type of Defect: Predominantly Bone (Bn), Cartilage (C), Mixed Both (Bo), Indirectly (IN), Neither (N)	Onset of disease age / diagnosis age	Genetic Changes	Life quality and length expectancy	Prevalence per 1,000,000 live births	Prevalence and category common / rare	Treatment	Plastic Surgery Involvement	Specific Procedures	Type of PRS Sx: Functional (Improving or restoring a physiological function (e.g., breathing, speaking, eating, vision), Aesthetic (primarily focused on improving physical appearance, though they may have psychological and social impacts excluding scar re	References
**22q11.2 Deletion Syndrome/Di George anomaly**	SYN	Bo	Birth/early childhood Clinical evaluation, genetic testing	*PITX2/TBX1, COMT, Cxcr4/Cxcl12; Sdf1, HIRA, UFD1L, CRKL, TBX1 TCOF1, POLR1C, POLR1D*	Quality of life varies widely depending on the severity and combination of symptoms. Early interventions, regular medical care, and supportive therapies (speech, occupational, and physical therapy) can significantly improve outcomes.	0.00020000	Life expectancy can be reduced due to congenital heart defects and immune deficiencies, but with appropriate medical care, many individuals can live into adulthood.	Medical Management:<br><br>Cardiac Defects: Surgical repair of heart anomalies. Immune Deficiency: Monitoring and management of infections, possibly thymus transplantation. Hypocalcemia: Calcium and vitamin D supplementation. Developmental Delays: Early intervention programs,	Cleft Palate Repair: Many children with 22q11.2 Deletion Syndrome have cleft palate or velopharyngeal insufficiency, requiring surgical intervention. Facial Reconstruction: For anomalies such as micrognathia (small jaw) or other craniofacial abnormalities. Ongoing Care: Plastic surgeons may also be involved in the management of scar tissue and secondary surgeries to improve functional and aesthetic outcomes.	Required:<br>Cleft Palate Repair<br>Velopharyngeal Insufficiency (VPI) Surgery<br>Jaw Surgery (Mandibular or Maxillary Osteotomies)<br>Nasal Reconstruction<br><br>Non CFS/Recommended.<br>Cardiac Surgery (if associated cardiac defects are present)<br>Facial Asymmetry Correction<br>Ear Reconstruction	Mixed	(3, 20, 33, 68, 79-86, 89)

								special education services, and therapies.				
**3MC Syndrome / Malpuech-Michels-Mingarelli-Carnevale syndrome**	CFS	Bn	Diagnosed by genetic testing, clinical evaluation	*COLEC11, MASP1/3*	Impact: developmental delays, physical anomalies	0.00000100	Surgery for physical anomalies, supportive therapies	Variable, depends on severity of anomalies and associated issues	Facial reconstruction, correction of craniofacial anomalies. The specific plastic surgery procedures commonly associated with 3MC syndrome include:	Functional<br>Cranial vault remodeling<br>Cleft L/P Repair<br>Midface Advancement (Le Fort)<br>Mandibular Osteotomies (Jaw Sx)<br><br>Aesthetic<br>Orbital Reconstruction<br>Eyelid, Lacrimal and Nasal Recon	Mixed	(132-134)
**[The] 6p25 deletion syndrome/Axenfeld-Rieger Syndrome**	CFS	Bo	Diagnosed by genetic testing Clinical evaluation, genetic testing	*FOXC1, PITX2, CYP1B1, PRDM5, PAX6*	Impact: developmental delays, physical anomalies Impact: vision issues, dental problems, regular monitoring	0.00000500	Variable, depends on severity of anomalies and associated issues Generally normal with proper management	Surgery for physical anomalies, supportive therapies Surgical correction of ocular anomalies, dental treatment	Correction of craniofacial and ocular anomalies Correction of ocular and dental anomalies These procedures are aimed at improving both function (vision, breathing, and dental occlusion) and appearance, ensuring that patients with ARS can lead healthier lives with fewer complications related to their craniofacial abnormalities.	Functional<br>Jaw Surgery (Mandibular or Maxillary Osteotomies). Required when there is significant jaw misalignment or underdevelopment (e.g., mandibular/maxillary hypoplasia) affecting breathing, chewing, or dental occlusion.<br>Cranial Vault Remodeling. Required in cases of craniosynostosis, where premature fusion of skull bones can lead to increased intracranial pressure and abnormal skull development, potentially impacting brain growth.<br>Cleft Lip and Palate Repair. Required if present, as cleft lip/palate affects feeding, speech, and can increase the risk of infections and other complications.<br>Nasal Reconstruction (Functional). Required if there are breathing difficulties due to nasal malformations (e.g., deviated septum). A septoplasty or other nasal reconstruction would be	Mixed	(135-138)

										performed to restore normal airflow. Midface Advancement (Le Fort Procedures)<br><br>Recommended (but Optional) Procedures These surgeries are generally recommended to improve aesthetics or enhance quality of life but are not critical to overall function or health. Orbital Reconstruction (Aesthetic). Eyelid and Orbital Reconstruction. Required if ptosis (drooping eyelids) or other eyelid malformations impair vision. Required if there are structural orbital abnormalities that need correction to support vision and eye function.		
**ADULT (Acro-Dermato-Ungual-Lacrimal-Tooth Syndrome) Syndrome**	CFS	In	Diagnosed by clinical evaluation and genetic testing	*TP63*	Impact: limb abnormalities, requires multidisciplinary care	0.00000100	Generally normal with proper management	Surgical correction of limb anomalies, supportive therapies	Correction of limb anomalies, skin abnormalities	Required<br>tear drainage (lacrimal system), dental function (dental reconstruction), chewing/speaking (jaw surgery), and skin health (skin grafting). Recommended. Correction of ectrodactyly, nail bed reconstruction, facial reconstruction, and scar revision.	Mixed	(63, 139)
**AEC Syndrome/Ankyloblepharon-Ectodermal defects-Clefting/ Hay-Wells syndrome**	CFS	In	Diagnosed by clinical evaluation and genetic testing	*TP63*	Impact: skin abnormalities, physical anomalies	0.00001429	Generally normal with proper management	Surgical correction of skin and mucous membrane anomalies	Correction of skin and mucous membrane anomalies	Required<br>Cleft Lip and Palate Repair<br>Ankyloblepharon Repair (Eyelid Surgery)<br>Skin Grafting and Wound Management<br><br>Rec.<br>Scar Revision<br>Dental Reconstruction<br>Nail Bed Reconstruction<br>Ear Reconstruction<br>Hair Transplantation or Scalp Reconstruction	Mixed	(63, 140, 141)

										Facial Contouring or Reconstruction		
**Alagille Syndrome**	SYN	Bn	Clinical evaluation, liver biopsy, and genetic testing	*JAGGED1, NOTCH2*	Impact: liver disease, heart defects, lifelong management	0.00002000	Variable, depends on severity of liver and heart defects	Liver transplant in severe cases, surgery for heart defects	Correction of craniofacial anomalies, repair of heart defects	Required<br>Eyelid ptosis repair and jaw surgery for mandibular hypoplasia, which are crucial for maintaining normal vision, dental function, and breathing<br><br>Recommended:<br>facial contouring, ear reconstruction, nasal reconstruction, and scar revision.	Mixed	(113, 142-144)
**Allgrove Syndrome/Triple-A syndrome (achalasia-addisonianism-alacrima syndrome)**	SYN	N	Clinical evaluation and genetic testing	*ALADIN (nuclear pore complex)*	Impact: multiple organ involvement, lifelong management	0.00000100	Reduced, variable depending on management of symptoms	Hormone replacement therapy, supportive care, regular monitoring	Correction of any physical anomalies	Required<br>Eyelid surgery for alacrima to prevent eye damage and esophageal surgery for achalasia<br><br>Recomm:<br>Scar revision, facial contouring, or managing skin changes from adrenal insufficiency.	Functional	(145-148)
**Aortic Aneurysm / Arches**	PT	N	Imaging studies (CT, MRI, ultrasound)	*Various genes including FBN1, TGFBR2*	Impact: depends on aneurysm size and location, risk of rupture	0.00010000	Reduced if aneurysm not managed properly	Surgical intervention for aneurysm repair, regular monitoring	Repair of associated craniofacial anomalies	"Aortic Aneurysm / Arches" generally refers to vascular conditions and does not directly involve craniofacial structures, bones, or cartilage. However, when these vascular abnormalities are part of genetic syndromes like Marfan syndrome, Loeys-Dietz syndrome, or Ehlers-Danlos syndrome, there can be associated craniofacial, skeletal, and connective tissue anomalies. These syndromes highlight the connection between the vascular system and other structural abnormalities in the body.	Neither	(16, 126, 127)
**Auriculo-Condylar Syndrome**	CFS	Bo	Clinical evaluation and	*PLCB4, GNAI3, EDN1,*	Impact: craniofacial abnormalities,	0.00000100	Generally normal with	Surgical correction of craniofacial	Correction of craniofacial	Mandibular Reconstruction (Mandibular Osteotomy)<br>Orthognathic Surgery	Mixed	(65, 149-152)

			genetic testing	*HDAC9, TWIST1*	multidisciplinary care		proper management	anomalies, supportive therapies	anomalies, ear reconstruction	Not PRS/Recomennded:<br>Airway Management (Tracheostomy or Jaw Advancement)<br>Otoplasty (Ear Reconstruction)<br>Facial Contouring<br>Secondary Mandibular Adjustments<br>Scar Revision<br>Nasal Reconstruction		
**Aplasia Cutis Congenita (ACC)**	PT	N	Clinical evaluation, genetic testing	*Various genetic and environmental factors KCTD1, KCTD15*	Impact: skin defects, potential for infection, surgical correction	0.00010000	Generally normal with proper management	Surgical correction of skin defects, supportive therapies	Correction of skin defects	Functional<br>Skin grafting and flap surgery<br><br>Aesthetic<br>Scar revision, tissue expansion, and aesthetic contouring	Aesthetic	(153)
**Bamforth-Lazarus Syndrome**	SYN	Bo	Clinical evaluation, genetic testing	*FOXE1*	Impact: thyroid dysfunction, hormone replacement therapy	0.00000100	Generally normal with proper management	Hormone replacement therapy, supportive care, regular monitoring	Correction of craniofacial anomalies	Functional<br>Jaw Surgery (Mandibular or Maxillary Osteotomy)<br>Choanal Atresia Repair<br>Thyroid Hormone Replacement (Endocrinological Treatment)<br><br>Aesthetic<br>Facial Contouring<br>Nasal Reconstruction (Rhinoplasty)<br>Otoplasty (Ear Surgery)<br>Scar Revision	Mixed	(154, 155)
**Bardet -Biedl Syndrome 8**	SYN	C	Diagnosis	*TTC8*	Quality of Life	0.00000667	Life Expectancy	Treatment	Plastic Surgery Involvement The most common surgical interventions are related to polydactyly correction and dental management. While craniofacial and cartilage defects are generally mild, plastic surgery may also	Functional<br>polydactyly correction, bariatric surgery, and kidney transplantation<br><br>Aesthetic<br>facial contouring, cosmetic body contouring, and scar revision.	Mixed	(156, 157)

									be needed for ear reconstruction or other facial corrections to improve function and appearance.			
**Binder Syndrome/ maxillonasal dysplasia or nasomaxillary hypoplasia Binder's Syndrome with Dysautonomia**	CFS	Bo	Clinical evaluation, genetic testing	*TTR, NEUROG1 (5q31.1)*	Impact: vision loss, kidney dysfunction, requires multidisciplinary care	0.00010000	Reduced, variable depending on severity of symptoms	Supportive care, management of symptoms, surgical interventions	Correction of facial anomalies, renal transplantation Binder Syndrome involves significant craniofacial abnormalities, particularly in the midface and nasal regions, due to underdevelopment of the maxilla and nasal structures. Plastic surgery plays a crucial role in managing Binder Syndrome, with key interventions including midface reconstruction (Le Fort I osteotomy or distraction osteogenesis), nasal reconstruction (rhinoplasty and septoplasty), and orthognathic surgery to correct dental malocclusion. These surgical procedures are essential for improving both the functional and aesthetic outcomes for individuals with	Functional<br>Midface reconstruction (Le Fort I osteotomy or distraction osteogenesis)<br>nasal reconstruction (rhinoplasty and septoplasty)<br>orthognathic surgery to correct dental malocclusions.<br>Palatal Expansion<br><br>Aesthetic<br>Secondary Rhinoplasty<br>Facial Contouring<br>Soft Tissue Augmentation<br>Scar Revision<br>"Functional<br>Maxillary Advancement Surgery (Le Fort I Osteotomy)<br>Nasal Reconstruction (Rhinoplasty)<br>Orthodontic Treatment<br>Airway Management (Considering Dysautonomia)<br><br>Aesthetic<br>Secondary Rhinoplasty<br>Facial Contouring<br>Scar Revision<br>Soft Tissue Augmentation	Mixed	(158-160)

									Binder Syndrome, enhancing their quality of life and self-esteem. significant craniofacial abnormalities, particularly midface hypoplasia and nasal deformities, alongside the challenges of Dysautonomia. Plastic surgery plays a key role in managing the structural issues of Binder Syndrome, with procedures like Le Fort I osteotomy, rhinoplasty, and orthognathic surgery being critical for correcting facial asymmetry, improving function, and enhancing aesthetics			
**Branchio-Oculo-Facial Syn.**	CFS	Bo	Clinical evaluation, genetic testing	*TFAP2A*	Impact: craniofacial abnormalities, requires surgical correction	0.00000100	Generally normal with proper management	Surgical correction of craniofacial anomalies	Branchio-Oculo-Facial Syndrome (BOFS) involves a combination of craniofacial anomalies, ocular defects, and skin abnormalities, with significant impacts on both appearance and function. Plastic surgery plays a critical role in managing the cleft lip/palate,	Functional<br>Branchial Cleft Cyst Excision<br>Cleft Lip and Palate Repair<br>Nasolacrimal Duct Surgery<br>Skin Defect Management (Alopecia and Erosions)<br><br>Aesthetic<br>Eyelid Surgery (Blepharoplasty), Facial reconstruction, rhinoplasty, otoplasty, scar revision, and orthodontic treatmen	Mixed	(161-163)

									branchial cleft anomalies, ear and nasal deformities, and other facial dysmorphisms associated with BOFS.			
**BOR (Branchio-Oto-Renal) Syndrome/Melnick-Fraser syndrome**	CFS	Bo	Clinical evaluation, genetic testing	*EYA1, SIX1, SIX5*	Impact: hearing loss, kidney dysfunction, requires multidisciplinary care	0.00002500	Variable, depends on severity of symptoms	Supportive care, surgical correction of anomalies	Correction of ear, neck, and facial anomalies involves craniofacial, ear, and renal anomalies. Plastic surgery plays a crucial role in managing the structural abnormalities associated with BOR syndrome, particularly in removing branchial cleft anomalies, reconstructing malformed ears, and addressing facial asymmetry.	Functional<br>Branchial Cleft Cyst/Fistula Excision<br>Ear Surgery (Otoplasty or Reconstructive Surgery)<br><br>Not PRS/Aesthetic<br>Hearing Restoration Surgery (Tympanoplasty or Cochlear Implantation)<br>Kidney Surgery (if renal abnormalities are severe)<br>Facial Contouring or Reconstruction<br>Scar Revision<br>Secondary Otoplasty (Ear Refinement)<br>Soft Tissue Augmentation	Mixed	(164-168)
**Charcot-Marie-Tooth**	SYN	N	Clinical evaluation, genetic testing	*PMP22, MPZ, LITAF, EGR2 and NEFL, sox10, cx32*	Impact: muscle weakness, loss of sensation	0.00040000	Generally normal with proper management	Supportive care, physical therapy, orthotics	Hand/Foot Recon "Orthopedic Surgery:<br>Foot Reconstruction:<br>In cases of severe foot deformities (e.g., pes cavus, hammer toes), orthopedic surgery may be necessary to correct the structure of the foot and improve mobility. This could involve tendon transfers, osteotomies (bone cutting), or	Orthopedic Surgery:<br>Foot Reconstruction: In cases of severe foot deformities (e.g., pes cavus, hammer toes), orthopedic surgery may be necessary to correct the structure of the foot and improve mobility. This could involve tendon transfers, osteotomies (bone cutting), or fusion of joints to stabilize the foot.<br>Hand Surgery:<br>In advanced cases where hand weakness and deformities are significant, surgical interventions may be necessary to improve hand function. This could	Functional	(40, 169, 170)

									fusion of joints to stabilize the foot.<br>Hand Surgery: In advanced cases where hand weakness and deformities are significant, surgical interventions may be necessary to improve hand function. This could include tendon transfer surgeries to restore grip strength and function."	include tendon transfer surgeries to restore grip strength and function.		
**Craniosynostosis**	CFS	Bn	Clinical evaluation, imaging studies	*FGFR2, TWIST1 SOX9, FGFR2, FGFR3, TWIST1, MSX2, EFNB1, POR, RAB23, RECQL4, GLI3*	Impact: craniofacial abnormalities, requires surgical correction	0.00044444	Generally normal with proper management	Surgical correction of skull sutures	Correction of craniofacial anomalies	Functional<br>Cranial Vault Remodeling<br>Frontoorbital Advancement (FOA) (if craniosynostosis)<br><br>Aesthetic<br>facial recon<br>ear/eye recon	Functional	(1-3, 5, 78, 79, 171)
**CHARGE Syndrome (Coloboma (eye defects), Heart defects, Atresia of the choanae (blockage of the nasal passages), Retarded growth and development**	SYN	Bo	Clinical evaluation, genetic testing	*CHD7*	Impact: multiple organ involvement, requires multidisciplinary care	0.00011111	Variable, depends on severity of symptoms	Supportive care, surgical correction of anomalies	Correction of craniofacial anomalies CHARGE syndrome is a complex genetic disorder with multiple congenital anomalies, including craniofacial abnormalities, heart defects, choanal atresia, and hearing loss. The syndrome involves significant bone	Functional<br>Choanal Atresia Repair<br>Facial Reanimation<br>Heart Surgery (for Congenital Heart Defects)<br>Tracheostomy and Airway Management<br>Gastrostomy Tube (G-Tube) Placement<br>Ocular Surgery (Coloboma Repair or Cataract Removal)<br>Ear Surgery (Cochlear Implants or Otoplasty)<br>FOA (midface hypoplasia)<br>Mandibular recon (jaw deformities)<br><br>Aesthetic	Mixed	(67, 69, 70)

									and cartilage defects, particularly in the ears, nose, and facial structures. Plastic surgery plays a crucial role in managing these defects, with interventions such as *minor * cleft lip/palate repair, choanal atresia surgery, ear reconstruction, and craniofacial surgery being essential for improving function and appearance.	Facial Contouring for asymmetry		
**Orofacial Cleft Orofacial cleft 5 (OFC5, MSX1 predominance)**	CFS	Bo	Clinical evaluation, imaging studies	*Various genetic and environmental factors, MSX1*	Impact: speech and feeding difficulties, requires surgical correction	0.00142857	Generally normal with proper management	Surgical correction of cleft lip and palate	Correction of cleft lip and palate<br><br>Surgical intervention for cleft lip and palate typically begins in infancy. Cleft lip repair is performed around 3-6 months of age, while cleft palate repair is usually done between 9-18 months of age. Alveolar bone grafting and orthodontic treatment are often performed in childhood or adolescence, while nasal reconstruction and jaw surgery	Functional<br>Cleft Lip Repair (Cheiloplasty)<br>Cleft Palate Repair (Palatoplasty)<br>Alveolar bone grafting<br><br>Aesthetic<br>Nasal recon, scar revision, jaw surg	Functional	(61, 172-174)

									are usually delayed until the face has finished growing in late adolescence or early adulthood.		
**Cochleovestibular Nerve Malformations**	CO	In	Clinical evaluation, imaging studies	*SOX10, EDNRB, GJB2*	Impact: hearing loss, balance issues, requires multidisciplinary care	0.00400000	Generally normal with proper management	Supportive care, surgical correction	Correction of auditory and vestibular anomalies	Functional<br>cochlear implantation, hearing aids, vestibular rehabilitation therapy, and speech therapy for hearing and language development. In cases of vestibular schwannoma, surgical tumor removal<br><br>Aesthetic<br>bone-anchored hearing aids, facial nerve monitoring, and tinnitus management	Neither	(168, 175)
**Congenital Central Hypoventilation Syndrome (Ondine's Curse)**	PT	N	Clinical evaluation, genetic testing	*RET, GDNF Dermatan sulfate pathway mutations Deletion at 6p25 region EDN3, TRK/BDNF, PHOX2A and B, GFRA1, BMP2, ECE1*	Impact: breathing difficulties, requires ventilatory support	0.00000500	Reduced, requires lifelong management	Ventilatory support, regular monitoring	Not typically involved	N/A	Neither	(21, 24, 31, 52-55)
**Congenital Pupillary-Iris-Lens Membrane with Goniodysgenesis**	CO	N				0.00000100			Plastic surgery is not typically involved in the direct treatment of this condition but may be considered if there are associated external ocular or facial anomalies	Functional<br>Surgical Membrane Removal (Pupillary Membranectomy)<br>Lens Surgery (Cataract Removal or Lens Replacement)<br><br>Aesthetic<br>Laser iridotomy, secondary lens procedures, corneal surgery	Neither	(53, 117, 176, 177)
**Cranioectodermal dysplasia/Sensenbrenner syndrome**	CFS	Bo	Clinical evaluatio	*WDR35, IFT122, WDR19,*	Impact: craniofacial abnormalities,	0.00000100	Variable, depends	Supportive care, surgical	Correction of craniofacial anomalies	Functional<br>Cranial vault remodeling	Mixed	(77, 89, 91, 92, 178)

			n, genetic testing	*IFT43, IFT22*	kidney and liver issues		on severity of sympto ms	correction of anomalies		Not PRS/Aesthetic kidney management for renal disease, dental interventions, respiratory support, and liver management. orthopedic surgery, facial contouring, and ophthalmologic interventions		
**Craniofacial-Deafness-Hand Syndrome**	SYN	Bo	Clinical evaluatio n, genetic testing	*PAX3*	Impact: craniofacial abnormalities, hearing loss	0.00000 100	Generall y normal with proper manage ment	Surgical intervention for Craniofacial-Deafness-Hand Syndrome is typically staged based on the patient's age and development. Cochlear implants or hearing aids are often placed early in life to optimize speech and language development, while craniofacial surgery and hand surgery are usually performed in childhood or adolescence, depending on the severity of the abnormalities. Orthognathic surgery and facial contouring	Correction of craniofacial anomalies significant bone and cartilage defects, particularly affecting the face and hands. Plastic surgery is essential in managing these structural anomalies, with procedures aimed at correcting craniofacial deformities, improving hand function, and restoring hearing.	Functional<br>Cochlear Implants or Hearing Aids<br>Craniofacial reconstruction or Le Fort osteotomy for midface hypoplasia.<br>Hand Surgery (Syndactyly or Polydactyly Correction)<br>Jaw Sx for micrognathia<br><br>Aesthetic<br>Facial contouring<br>Otoplasty	Mixed	(39, 47, 179)

								are typically delayed until the patient has reached skeletal maturity in late adolescence.				
**Craniofacial anomalies associated with Kearns-Sayre Syndrome**	SYN	In	Clinical evaluation, genetic testing	*RET, EDNRB, GDNF, SOX10, PHOX2B*		0.00002000			Kearns-Sayre Syndrome is a mitochondrial disorder primarily affecting the eyes, muscles, and heart, with some associated craniofacial anomalies such as ptosis, facial muscle weakness, and midfacial hypoplasia.	Functional<br>Cardiac conduction defects, ptosis affecting vision, ocular issues, and endocrine abnormalities. These include pacemaker placement, ptosis surgery, and management of retinal degeneration and hearing loss.<br><br>Aesthetic<br>low vision aids, physical therapy, and cosmetic surgery	Neither	(22-25, 180)
**CSC-KT (Cardiofaciocutaneous syndrome, Keratosis Follicularis Spinulosa Decalvans (KFSD), and Tangier disease)**	SYN	Bo	Clinical evaluation, genetic testing	*BRAF, MAP2K1, MAP2K2, MAPRE2*		0.00000123				Functional<br>Le Fort Osteotomy<br>Mandibular Advancement (Orthognathic Surgery)<br>Fronto-Orbital Advancement<br><br>Aesthetic<br>Skin	Mixed	(5, 181)
**Diamond Blackfan Anemia Syn.**	SYN	Bo	Clinical evaluation, genetic testing	*At least 14 Ribosomal proteins including: RPL5, RPL11, RPS19*	Impact: anemia, requires regular blood transfusions	0.00000600	Variable, depends on severity of symptoms	Regular blood transfusions, corticosteroids	Bone and Cartilage Defects: Craniofacial Abnormalities: These include midface hypoplasia, high-arched palate, and occasionally micrognathia. The cranial bones may be affected, leading to abnormal facial growth and structure. Cartilage	Functional<br>Cleft Lip and Palate Repair:<br>Thumb Reconstruction (Pollicization)<br>midface hypoplasia, and occasionally micrognathia. repair.<br><br>Aesthetic<br>Scar revision<br>Soft tissue aug<br>Otoplasty	Functional	(100, 182)

									Involvement: Ear cartilage may be underdeveloped or abnormally shaped, contributing to the low-set, posteriorly rotated ears. Plastic Surgery Involvement: Craniofacial Surgery: Procedures to address midface hypoplasia, including orthognathic surgery or Le Fort osteotomies, may be necessary to correct facial asymmetry and improve function. Otoplasty: Reconstructive ear surgery might be performed to correct the shape and positioning of the ears. Scar and Skin Treatment: Treatment of skin issues, such as hyperkeratosis, might involve laser therapy or topical treatments. Surgery for scar revision may also be considered if necessary.			
**EEC3 Syndrome**	CFS	Bo	Clinical evaluation, genetic testing	*TP63*	Impact: ectodermal dysplasia, cleft lip and palate,	0.00001111	Generally normal with proper	Surgical correction of cleft lip and palate,	Correction of cleft lip and palate, limb anomalies	Cleft Lip and Palate Repair Hand/Foot Recon	Functional	(63, 101, 183, 184)

					limb abnormalities		manage ment	supportive care				
**Ellis-van Creveld syndrome**	SYN	Bn	Clinical evaluatio n, genetic testing	*EVC, EVC2*	Impact: skeletal abnormalities, heart defects, requires multidisciplinar y care	0.00000 769	Variable , depends on severity of sympto ms	Supportive care, surgical correction of anomalies	Correction of skeletal anomalies Polydactyly correction is usually performed during early childhood to allow normal hand and foot function as the child grows. Cleft palate repair is typically performed in infancy, while dental surgeries and orthodontic interventions occur as the teeth develop, usually in childhood and adolescence. Orthopedic surgeries may be delayed until the child reaches skeletal maturity, depending on the severity of the limb deformities.	Functional<br>Polydactyly Correction<br>Cleft Palate Repair<br>Hand Function Improvement<br><br>Aesthetic<br>Scar Revision<br>Orthopedic Surgery	Functional	(91, 185-187)
**Facial anomalies in cytochrome-oxidase deficiency (and subsequent Kearns-Sayre syndrome with growth hormone deficiency)**	CO	In	Clinical evaluatio n, genetic testing	*MITF, SNAI2, SOX10, EDNRB*	Impact: muscle weakness, heart block, growth hormone deficiency	0.00002 000	Variable , depends on severity of sympto ms	Supportive care, management of symptoms	Correction of craniofacial anomalies	Functional<br>Ptosis correction (levator resection or frontalis suspension)<br>Midface reconstruction (Le Fort osteotomy)	Aesthetic	(180)
**Frontonasal Dysplasia/median cleft face syndrome**	CFS	Bo	Clinical evaluatio n, imaging studies	*GLI1, ALX3 (FND1), ALX4 (FND2), ALX1 (FND3), EFNB1, Kif3a*	Impact: craniofacial abnormalities, requires surgical correction	0.00000 100	Generall y normal with proper manage ment	Surgical correction of craniofacial anomalies	Correction of craniofacial anomalies	Functional<br>Cranial Vault Remodeling (for cranial abnormalities):<br>Midface Reconstruction (for hypertelorism and clefts) / orbital osteotomy)<br>Nasal Reconstruction<br>Cleft Lip & Palate Repair	Functional	(115, 188, 189)

**Familial dysautonomia/Riley-Day syndrome**	PT	N	Clinical evaluation, genetic testing	' *ELP1*	Impact: autonomic dysfunction, requires multidisciplinary care	0.00003226	Reduced, variable depending on severity of symptoms	Supportive care, management of symptoms	Timing of Surgery: Gastrostomy tube placement may be performed early in life when feeding difficulties become apparent. Facial reanimation surgery and jaw surgery are typically performed later in childhood or adolescence, once facial growth is more complete and motor function limitations are fully assessed. Scar revision can be performed after previous surgeries have healed. Spinal fusion surgery is usually considered in adolescence when scoliosis becomes more pronounced.<br><br>Craniofacial Involvement:<br><br>Bone Defects: Craniofacial bone defects are not a primary feature of familial dysautonomia. However, some individuals may have facial asymmetry or other minor craniofacial	Functional Gastrostomy Tube (G-Tube) Placement<br><br>Recommended Procedures: Facial Reanimation Surgery<br>Sialorrhea Management<br>Orthognathic Surgery<br>Scar Revision	Neither	(190-192)

									anomalies related to the overall neurological dysfunction. Cartilage Defects: There are no specific cartilage defects associated with familial dysautonomia. The condition primarily affects the autonomic and sensory nervous systems rather than structural components like cartilage. Plastic Surgery Correction: Craniofacial Corrections: Plastic surgery is generally not a primary component of treatment for familial dysautonomia, as the condition does not typically involve significant craniofacial anomalies that would require surgical correction."			
**Fryns Syndrome with Hirschprung Disease**	SYN	Bo	Birth/early childhood Clinical evaluation	*PIGN*	Very high neonatal mortality rate, most infants are still born of die in the neonatal period dur to	.00007	rare	Post surgical bowel management for Hirschprung, surgical management of	Surgical correction of alveolar cleft		Functional	(193, 194)

					congenital diagphragmatic hernia and severe respiratory insufficiency. Presence of Hirschprung disease adds surgical and medical complexity with bowel obstruction and enterocolitis risk.			diagphragmatic hernia				
**Fusiform Aneurysm**	PT	N	Imaging studies (CT, MRI, ultrasound)	*Unknown*	Impact: depends on aneurysm size and location, risk of rupture	0.00001000	Reduced if aneurysm not managed properly	Surgical intervention for aneurysm repair, regular monitoring	There are no specific cartilage defects associated with fusiform aneurysms. The condition involves the arterial walls, which are composed of muscle and connective tissue rather than cartilage. Plastic Surgery Correction: Craniofacial Corrections: Plastic surgery is not typically involved in the treatment of fusiform aneurysms. However, if a cerebral aneurysm results in neurological deficits affecting the face (e.g., facial nerve palsy),	Fusiform aneurysms themselves do not directly cause craniofacial bone defects. However, large cerebral fusiform aneurysms may indirectly affect craniofacial structures if they compress cranial nerves or other adjacent structures within the skull.	Neither	(195)

									reconstructive surgery or rehabilitative procedures may be considered.			
**Giant Congenital Melanocytic Nevus**	PT	N	Clinical evaluation, imaging studies	*NRAS*	Impact: large pigmented moles, risk of melanoma, requires multidisciplinary care	0.00005000	Generally normal with proper management	Surgical removal of nevi, regular monitoring	Removal of large nevi, correction of associated anomalies.<br><br>Craniofacial Corrections: Excision: Surgical removal of the nevus is the primary treatment approach. Due to the size of GCMNs, excision often requires multiple stages and may involve complex reconstructive techniques to minimize scarring and maintain function. Tissue Expansion: This technique involves inserting an expander under the adjacent normal skin, which is gradually inflated over time to stretch the skin. The expanded skin is then used to cover the area where the nevus is excised. Skin Grafting: In cases where direct closure is not possible, skin	Functional<br>Nevus Excision.<br>Skin Grafting or Flap Surgery: Used to cover large defects left after nevus removal.	Aesthetic	(196-199)

									grafts may be used to cover the excised area. Full-thickness grafts are preferred for facial areas to achieve a better cosmetic outcome. Flap Reconstruction: Local or distant flaps may be employed to reconstruct the area after nevus removal, particularly when significant tissue is involved."			
**Goldberg–Shprintzen Megacolon Syndrome (GSMS)**	SYN	Bo	Clinical evaluation, genetic testing	*KIFBP, EYA1, SIX1, SIX5*	Impact: gastrointestinal issues, requires surgical correction	0.00000100	Variable, depends on severity of symptoms	Surgical correction of megacolon, supportive care	Craniofacial Involvement:<br><br>Bone Defects: Craniofacial bone defects are common in GOSHS, particularly in the form of facial dysmorphism and microcephaly. These defects contribute to the characteristic facial appearance of affected individuals. Cartilage Defects: Cartilage defects are not a primary feature of GOSHS, but the craniofacial abnormalities may involve the structure and development of	Functional<br>Cleft Palate Repair (Palatoplasty).<br>Craniofacial Reconstruction<br>Midface advancement/Le Fort osteotomy for midface hypoplasia or orbital surgery for wide-set eyes (hypertelorism).<br><br>Aesthetic<br>Orthognathic Surgery (Jaw Surgery) (functional)<br>Scar revision	Mixed	(29, 72)

									cartilage, particularly in the nose and ears.<br>Plastic Surgery Correction:<br><br>Craniofacial Corrections:<br>Facial Reconstruction:<br>Plastic surgery may be considered to address significant craniofacial anomalies, particularly if they affect function (e.g., breathing, feeding) or cause significant cosmetic concerns.<br>Strabismus Correction:<br>Ophthalmologic surgery might be necessary to correct strabismus and improve visual alignment, which can be important for visual development.			
**Goldenhar's syndrome/Oculo-Auriculo-Vertebral Syndrome**	CFS	Bo	congenita l<br>Onset at birth	*GSC, SALL1, HOXA2/ SKI, SF3B2*	Cases can be mild with normal LE and QOL vs severe cases with worse outcomes<br>Impact: craniofacial abnormalities, hearing loss, requires	0.00006 667	Generall y normal with proper manage ment	surgery, speech therapy, hearing aids, educational support<br>Surgical correction of craniofacial anomalies, hearing aids	Timing of Surgery:<br>Cleft palate repair is typically performed in early infancy, around 9-18 months of age, to promote normal speech and feeding development.<br>Craniofacial	Functional<br>Mandibular Distraction Osteogenesis:<br>Cleft Lip and Palate Repair (if present)/Cheiloplasty (lip repair) and palatoplasty (palate repair)<br>Orbital and Midface Reconstruction//Le Fort osteotomies.<br>Ear Reconstruction (Microtia or Anotia).	Functional	(21, 200)

					surgical correction				reconstruction and orthognathic surgery are usually performed later in childhood or adolescence, depending on the severity of the facial deformities and the patient's growth. Scar revision and soft tissue augmentation are generally performed after primary surgeries have healed, often in late childhood or adolescence.	Aesthetic Ear Reconstruction (Microtia or Anotia):		
**Gorlin-Goltz Syndrome/basal cell carcinoma syndrome**	CFS	Bo	Clinical evaluation, genetic testing	*PTCH1*	Impact: multiple basal cell carcinomas, jaw cysts, requires multidisciplinary care	0.00003226	Generally normal with proper management	Surgical removal of basal cell carcinomas, management of symptoms	Removal of basal cell carcinomas, correction of jaw cysts multiple basal cell carcinomas, jaw cysts, craniofacial anomalies, and skeletal abnormalities. Bone and cartilage defects primarily involve the jaw (due to keratocystic odontogenic tumors), ribs (bifid ribs), and craniofacial structures (frontal bossing, cleft lip/palate). Plastic surgery plays a crucial role in managing these manifestations, particularly in	Functional BCC. Excisional Surgery. Mohs micrographic surgery. Bone Grafting or Flap Surgery: For reconstructing jawbone defects caused by recurrent cyst removal. Skin grafting or flap surgery.<br><br>Recommended Procedures: Facial Reconstruction: To address facial asymmetries such as frontal bossing. Laser Treatment for BCCs: To treat small, superficial lesions and minimize scarring. Scar Revision: To improve the appearance of scars from multiple surgeries. Orthognathic Surgery: For correcting jaw deformities and malocclusion after jaw cyst removal. (fxn)	Functional	(201, 202)

									the surgical removal of basal cell carcinomas, craniofacial reconstruction, and the management of skeletal abnormalities. Timing of Surgery: Excisional surgery for BCCs is performed as needed, starting from adolescence or early adulthood, depending on the onset of the lesions. Reconstructive surgery following tumor removal is typically done immediately after excision, while more complex procedures like facial reconstruction or jaw surgery may be performed later in childhood or adulthood based on growth and development.			
**Growth Retardation, Developmental Delay, And Facial Dysmorphism**	CFS	Bo	Clinical evaluation, genetic testing	*FTO*	Impact: growth and developmental delays, requires multidisciplinary care	0.00000100	Variable, depends on severity of symptoms	Supportive care, management of symptoms	Correction of craniofacial anomalies	Functional<br>Cleft Lip and Palate Repair<br>Mandibular Distraction Osteogenesis<br>Ear Reconstruction (Microtia or Malformed Ears)<br><br>Recommended Procedures:<br>Facial Recontouring.<br>Scar Revision.<br>Orthognathic Surgery. (fx)	Mixed	(203, 204)

**Hajdu-Cheney Syndrome**	SYN	Bn	Clinical evaluation, genetic testing	*NOTCH2*	Impact: skeletal abnormalities, requires surgical correction	0.00001000	Reduced, variable depending on severity of symptoms	Surgical correction of skeletal anomalies, supportive care	Correction of skeletal anomalies, craniofacial anomalies	Functional Mandibular Reconstruction/bone grafting Orthognathic Surgery (Jaw Surgery). Craniofacial Reconstruction.	Mixed	(111, 112, 114)
**Haddad syndrome (Ondine-Hirschsprung syndrome)**	PT	N	Clinical evaluation, genetic testing	*MTTL1, MTTL2, PHOX2B*	Impact: breathing and bowel difficulties, requires ventilatory support	0.00000100	Variable, depends on severity of symptoms	Surgical correction of heart and abdominal anomalies	Correction of associated craniofacial anomalies	Chest Wall Reconstruction: Functional/Aesthetic (for chest deformities). Facial Reconstruction (if craniofacial anomalies are present): Aesthetic (with functional benefits in some cases). Cleft Lip/Palate Repair: Functional (for feeding, speech, and aesthetics). Scar Revision: Aesthetic (for improving the appearance of surgical scars).	Neither	(31, 205, 206)
**Hirschsprung**	PT	N	Clinical evaluation, biopsy	*RET, GDNF, NRTN, NTN, SOX10, EDNRB, EDN3, ECE1, ZFHX1B, PHOX2B, KIAA1279, TCF4, NRG1, MAPK10, ZFHX1B, SOX2, L1CAM, -DNMT3B SIP1, HASH1, HAND2, PATCH1/NOTCH*	Impact: bowel obstruction, requires surgical correction	0.00020000	Generally normal with proper management	Surgical correction of bowel obstruction	Not typically involved	Perineal Reconstruction: Functional/Aesthetic (for complications affecting the perineum post-surgery). Abdominal Wall Scar Revision: Aesthetic (to minimize visible scarring). Colostomy Site Closure and Reconstruction: Functional/Aesthetic (to restore abdominal wall integrity and appearance). Anal Reconstruction: Functional (to restore normal bowel function if complications arise).	Neither	(21, 24, 29-36, 39, 43, 48, 50, 61, 71-76)
**Hypogonadotropic Hypogonadism/K**	PT	N	Clinical evaluation,	*FGF8, PROK2, PROKR2,*	Impact: delayed or absent puberty,	0.00001724	Generally normal with	Hormone replacement therapy,	Summary: Bone Defects: Yes, including	Gynecomastia Surgery (Males): Aesthetic and Functional (to correct	Neither	(46, 48-51, 68, 78, 207)

**allmann Syndrome**			hormonal testing	*CHD7, WDR11, FGFR1 KAL1, FGFR1, PROKR2, SOX10*	requires hormone replacement		proper manage ment	supportive care	osteopenia/osteo porosis and cleft lip/palate. Cartilage Defects: Yes, primarily associated with nasal cartilage abnormalities in cases with craniofacial anomalies. Plastic Surgery Involvement: Yes, particularly in craniofacial surgery (cleft lip/palate repair, rhinoplasty) and potentially in reconstructive surgery for associated anomalies. Kallmann Syndrome involves a combination of hypogonadotropi c hypogonadism and anosmia, with potential craniofacial anomalies that may require surgical correction. Plastic surgery is integral in managing these anomalies to improve function, appearance, and overall quality of life. Hormonal management and multidisciplinary care are also essential in addressing the	enlarged male breast tissue). Genital Reconstruction (Males): Functional and Aesthetic (for penile lengthening and orchiopexy). Breast Augmentation (Females): Aesthetic (to improve breast development). Rhinoplasty (if craniofacial anomalies are present): Aesthetic (to improve nasal symmetry).		

									broader impacts of the syndrome.			
**Hypohidrotic Ectodermal Dysplasia**	CFS	Bn	Clinical evaluation, genetic testing	*EDA1, EDAR, EDARADD, and WNT10A*	Impact: inability to sweat, dental abnormalities, requires multidisciplinary care	0.00005882	Generally normal with proper management	Supportive care, management of symptoms	Correction of dental and craniofacial anomalies	Dental Implants and Prosthetics: Functional and Aesthetic (for chewing, speech, and appearance). Facial Reconstruction: Functional and Aesthetic (for improving midface structure, nasal function, and appearance). Hair Restoration: Aesthetic (to restore scalp hair and improve appearance). Lip Augmentation: Aesthetic (to improve the appearance and proportion of lips). Scar Revision: Aesthetic (to improve the appearance of surgical scars).	Aesthetic	(63, 96, 208-210)
**Johnson-McMillin Syndrome**	SYN	Bo	Symptos/ diagnosis usually start in childhood	*unknown*	Impact: Issues affecting daily life include alopecia, anosmia or hyposmia, conductive hearing loss, microtia, craniofacial anomalies and delayed puberty or hypogonadism	0.000001	Life expectancy appears normal with patients surviving into adulthood.	Management includes audiology/ENT care for hearing loss, endocrine/reproductive management for hypogonadotropic hypogonadism	Yes, often when microtia or other craniofacial anomalies are present.	Auricular reconstruction/prosthetic ear, atresia/hearing reconstruction or other hearing rehabilitation, cleft palate repair when present.	Mixed	(211)
**JS-X syndrome**	CFS	Bo	Clinical evaluation, genetic testing	*Complex multifactorial, several genes implicated*	Impact: craniofacial abnormalities, requires surgical correction	0.00000100	Generally normal with proper management	Surgical correction of craniofacial anomalies	Correction of craniofacial anomalies	Facial Reconstruction: Functional and Aesthetic (for correcting craniofacial asymmetry, nasal defects, and midface development). Orthognathic Surgery (Jaw Surgery): Functional and Aesthetic (to correct jaw misalignment, improve chewing, and enhance facial symmetry). Gynecomastia Surgery: Aesthetic and Functional (to correct enlarged male breast tissue). Scar Revision: Aesthetic	Mixed	(212)

										(to improve the appearance of scars after surgery).		
**Kabuki Syndrome**	SYN	Bo	Clinical evaluation, genetic testing	*KMT2D, KDM6A*	Impact: developmental delays, growth retardation, craniofacial anomalies	0.00003125	Generally normal with proper management	Supportive care, surgical correction of anomalies	Correction of craniofacial anomalies	Cleft Palate Repair: Functional and Aesthetic (to restore speech, feeding, and appearance). Ear Reconstruction: Aesthetic (to improve ear appearance and facial symmetry). Facial Reconstruction: Aesthetic and Functional (to improve facial symmetry and potentially breathing/feeding). Hand Surgery: Functional (to correct finger anomalies for improved hand function). Scar Revision: Aesthetic (to reduce the appearance of surgical scars).	Mixed	(5, 119, 213-215)
**Klippel-Feil Syndrome**	SYN	Bo	Clinical evaluation, imaging studies	*MEOX1, GDF3, GDF6, MYO18B*	Impact: limited neck movement, requires surgical correction	0.00002439	Generally normal with proper management	Surgical correction of vertebral anomalies, supportive care	Bone Defects: Yes, including cervical vertebral fusion, scoliosis, rib anomalies, and potential craniofacial bone abnormalities. Cartilage Defects: Yes, potentially involving ear cartilage and, less commonly, tracheal cartilage. Plastic Surgery Involvement: Yes, particularly in craniofacial surgery (cleft palate repair, orthognathic surgery), scar revision, and possibly ear reconstruction.	Cervical Spine Reconstruction: Functional (for managing soft tissue defects after spinal surgery). Ear Reconstruction: Functional and Aesthetic (to improve hearing and ear appearance). Facial Reconstruction: Aesthetic and Functional (for correcting craniofacial deformities and improving appearance). Scar Revision: Aesthetic (to reduce scarring after surgeries).	Mixed	(66, 216-220)
**Lattice corneal dystrophy**	CO	N	first or second	*TGFBI*	LE normal, QOL can be	0.00001000		lubricating eye drops,	not involved	Corneal Transplant (Penetrating Keratoplasty	Neither	(221-224)

			decade of life		negatively impacted by vision impairment/pain			contact lenses, corneal transplant		or DALK): Functional (to restore vision). Ocular Surface Reconstruction: Functional (to repair the cornea and ocular surface). Eyelid Surgery (for Ectropion): Functional and Aesthetic (to correct eyelid malposition and reduce irritation).		
**Leigh syndrome**	PT	N	infancy/childhood	*SURF1, MT-ATP6*	significantly reduced, many do not survive childhood	0.00002500		supportive care, symptom managmeent, nutritional support, respiratory assistance, targeted therapies for genetic mutations	not involved	Contracture Release Surgery: Functional (to improve mobility). Scoliosis Surgery: Functional (to correct spinal curvature and improve posture). Facial Reconstruction: Aesthetic and Functional (if facial anomalies are present).	Neither	(225-227)
**Limb-Mammary**	PT	Bn	Clinical evaluation, genetic testing	*TP63*	Impact: limb and mammary gland abnormalities, requires surgical correction	0.00001000	Generally normal with proper management	Surgical correction of limb and mammary gland anomalies	Summary: Bone Defects: Yes, including limb malformations (ectrodactyly, hypoplasia) and cleft lip/palate. Cartilage Defects: Yes, potentially involving nasal and ear cartilage in association with craniofacial anomalies. Plastic Surgery Involvement: Yes, particularly in craniofacial surgery (cleft lip/palate repair, rhinoplasty), limb reconstruction, and breast reconstruction. Limb-Mammary	Hand and Foot Reconstruction (for Ectrodactyly): Functional and Aesthetic (to improve limb function and appearance). Breast Reconstruction (for Mammary Hypoplasia): Aesthetic (to improve breast development and symmetry). Nail Bed Reconstruction: Aesthetic (to improve the appearance of the nails). Lacrimal gland	Functional	(63, 101, 102)

									Syndrome requires a multidisciplinary approach to address the various physical anomalies. Plastic surgery is crucial in managing the craniofacial, limb, and breast abnormalities associated with the syndrome, aiming to improve function, appearance, and overall quality of life for affected individuals.<br><br>Craniofacial Involvement:<br>Bone Defects:<br>Craniofacial bone defects are not a primary feature of LMS, but secondary craniofacial anomalies, such as cleft lip/palate, may be present in some cases.<br>Cartilage Defects:<br>There are no specific cartilage defects directly associated with LMS. However, the malformations in the limbs and mammary glands may involve underlying ectodermal tissue, which can include cartilage			

									in some cases. Plastic Surgery Correction: Craniofacial Corrections: Cleft Lip/Palate Repair: For individuals with LMS who have a cleft lip and/or palate, plastic surgery is essential for repairing these defects. This typically involves multiple surgeries throughout childhood to ensure proper function and aesthetics<br><br>Summary of Clinical Features Defect Frequency Hand anomalies 18/27. Foot anomalies 21/27. Mammary gland hypo/aplasia 10/11 Nipple hypo/aplasia 27/27. Lacrimal-duct atresia 7/15. Nail dysplasia 7/21. Hypohydrosis 7/21. Cleft palate/bifid uvula 6/22 Hypodontia 3/21/ Furunculosis/hydradentitis 3/21. Earpits 2/21. Skin and/or hair anomalies 0/21			

**Lujan-Fryns Syndrome**	SYN	Bo		*MED12, UPF3B,*		0.00000667			Summary: Bone Defects: Yes, including Marfanoid habitus (long limbs, scoliosis, pectus excavatum) and high, narrow palate. Cartilage Defects: Yes, involving joint hypermobility and nasal cartilage abnormalities. Plastic Surgery Involvement: Yes, particularly in craniofacial surgery (palate repair, orthognathic surgery), orthopedic surgery (scoliosis and chest wall correction), and reconstructive surgery. Lujan-Fryns Syndrome is a complex genetic disorder that requires a multidisciplinary approach to address the various physical, intellectual, and behavioral challenges. Plastic surgery can play a crucial role in managing the craniofacial and skeletal anomalies associated with the syndrome, helping to	Orthognathic Surgery (Jaw Surgery): Functional and Aesthetic (to correct micrognathia and improve chewing and speech). Ear Reconstruction (Otoplasty): Aesthetic (to correct large, protruding ears and improve facial balance). Palate Surgery (for high-arched palate): Functional (to improve speech and dental alignment). Facial Contouring or Chin Augmentation: Aesthetic (to correct small chin and improve facial symmetry). Scar Revision: Aesthetic (to reduce visible scars from previous surgeries).	Mixed	(228-232)

									improve function, appearance, and overall quality of life for affected individuals.			
**Meckel-Gruber Syndrome**	SYN	Bn	Clinical evaluation, imaging studies	*MKS1, TMEM216, TMEM67, CEP290, RPGRIP1L, CC2D2A, NPHP3, TCTN2, B9D1, B9D2, TMEM231, KIF14, TMEM107, TXNDC15*	Impact: multiple organ involvement, requires multidisciplinary care	0.00000789	Reduced, variable depending on severity of symptoms	Supportive care, surgical correction of anomalies	Craniofacial Involvement:<br><br>Bone Defects: Cranial Bone Defects: The most significant craniofacial involvement in MKS is the presence of an encephalocele, where a defect in the cranial bones allows brain tissue to herniate outside the skull. Cartilage Defects: There are no specific cartilage defects directly associated with MKS, but craniofacial anomalies may involve soft tissue structures, including cartilage, particularly in the ears and nose. Plastic Surgery Correction:<br><br>Craniofacial Corrections: Encephalocele Repair: In rare cases where the encephalocele is small and the infant survives, surgical repair	Encephalocele Repair: Functional (to protect the brain and close the skull defect). Cleft Lip/Palate Repair: Functional and Aesthetic (to restore feeding, speech, and appearance). Polydactyly Correction: Functional and Aesthetic (to improve hand/foot function and appearance). Craniofacial Reconstruction: Functional and Aesthetic (to correct skull deformities and improve craniofacial symmetry).	Neither	(90, 93, 233-235)

									may be attempted to place the brain tissue back into the skull and close the defect. However, the overall prognosis is generally poor due to the severity of associated anomalies. Cleft Lip/Palate Repair: If an infant with MKS survives beyond the neonatal period, cleft lip/palate repair may be considered to improve feeding and speech development. However, this is rare given the typically lethal nature of the syndrome.			
**Medullary Thyroid - Carcinomas**	PT	N	Clinical evaluation, genetic testing	*MEN1, RET, CDKN1B*	Impact: requires surgical removal of thyroid and supportive care	0.00003333	Generally normal with proper management	Surgical removal of thyroid, supportive care	Not typically involved	Neck Dissection and Tumor Excision: Functional and Aesthetic (to remove cancer and maintain neck mobility and appearance). Reconstruction After Thyroidectomy: Functional and Aesthetic (to restore normal neck contour and function). Facial Nerve Repair: Functional (to restore vocal cord or facial nerve function). Scar Revision: Aesthetic (to improve the appearance of post-surgical scars). Flap Reconstruction: Functional and Aesthetic	Neither	(56-60, 62)

										(to cover defects after extensive tumor removal).		
**MED13L Haploinsufficiency Syndrome (MRFACD / Asadollahi-Rauch Syndrome)**	CO	In	Present at birth	*MED13L*	-	0.00000100		Surgical correction of cardiovascular anomalies	very rare syndrome, plastic surgery not described at this time	Orthognathic Surgery (Jaw Surgery): Functional and Aesthetic (to correct micrognathia and improve chewing, speech, and facial symmetry).<br>Palate Surgery: Functional and Aesthetic (to restore oral function and improve appearance in cases of cleft palate).<br>Dental Reconstruction: Functional and Aesthetic (to correct dental anomalies and restore proper oral function).<br>Facial Contouring or Rhinoplasty: Aesthetic (to improve craniofacial features such as nasal bridge and symmetry).<br>Scar Revision: Aesthetic (to reduce scarring after surgical interventions).	Neither	(236-238)
**Melanoma**	PT	In	Clinical evaluation, biopsy	*BRAF/NRAS, SOX10, MITFA, Trk/NT, CDKN2A, MCIR, CDK4, BRN3A, XRCC3, TERT, POT1*	Impact: skin cancer, requires surgical removal and monitoring	0.03030303	Variable, depends on stage at diagnosis	Surgical removal of melanoma, supportive care	Removal of melanoma, reconstruction of affected areas	Wide Local Excision: Functional and Aesthetic (removal of tumor with healthy tissue margin).<br>Sentinel Lymph Node Biopsy: Functional (assesses cancer spread).<br>Skin Grafting: Aesthetic and Functional (closes large defects after tumor excision).<br>Flap Reconstruction: Aesthetic and Functional (covers larger or complex defects, especially in the face or scalp).<br>Mohs Micrographic Surgery: Aesthetic and Functional (ensures complete removal while preserving healthy tissue).<br>Scar Revision: Aesthetic (reduces visible scarring after melanoma excision).<br>Facial Reconstruction:	Mixed	(239-242)

										Aesthetic and Functional (restores normal appearance and function after facial melanoma excision).		
**Mandibulofacial Dysostosis with Alopecia**	CFS	Bo	Clinical evaluation, genetic testing	*EDNRA*	Impact: craniofacial abnormalities, alopecia, requires surgical correction	0.00000100	Generally normal with proper management	Surgical correction of craniofacial anomalies	Correction of craniofacial anomalies, alopecia	Orthognathic Surgery (Jaw Surgery): Functional and Aesthetic (to correct mandibular hypoplasia and improve chewing, speech, and facial symmetry). Cleft Palate Repair (If Present): Functional and Aesthetic (to restore feeding, speech, and facial appearance). Ear Reconstruction (Microtia or Malformed Ears): Functional and Aesthetic (to restore ear function and appearance, and potentially improve hearing). Hair Restoration (For Alopecia): Aesthetic (to restore scalp hair and improve self-esteem). Facial Reconstruction: Functional and Aesthetic (to correct facial asymmetry and improve overall facial structure). Scar Revision: Aesthetic (to minimize the appearance of scars from previous surgeries).	Mixed	(243, 244)
**Median Cephalic Neurocristopathy**	CFS	Bo	Clinical evaluation, genetic testing	*Unknown*		0.00000100			Median cephalic neurocristopathy refers to a group of disorders that arise from defects in the development of neural crest cells, which contribute to the formation of the face, skull, and other structures. These disorders often result in midline	1. Cleft Lip and Palate Repair<br>Procedure: Repairing the cleft lip and palate is one of the primary interventions for individuals with midline clefts. Cheiloplasty (lip repair) and palatoplasty (palate repair) are performed to close the gaps, restore oral function, and improve speech development.<br>Procedure details:	Mixed	(245)

									facial defects.<br><br>Craniofacial Involvement:<br><br>Bone Defects: Craniofacial bone defects are common, potentially including midline facial clefts, underdeveloped midface, and abnormalities of the nasal and frontal bones.<br>Cartilage Defects: Cartilage defects may involve the nasal septum and other midline structures, depending on the specific presentation of the neurocristopathy.<br>Plastic Surgery Correction: Extensive plastic and reconstructive surgery is often required to correct the facial clefts, midface hypoplasia, and other craniofacial anomalies associated with median cephalic neurocristopathy.	Surgeons reconstruct the upper lip and close the gap in the roof of the mouth to enable normal feeding, speaking, and swallowing.<br>Categorization: Functional and Aesthetic — Restores feeding and speech ability, and improves facial appearance.<br>2. Nasal Reconstruction (Rhinoplasty)<br>Procedure: Patients with bifid noses or other nasal deformities may require nasal reconstruction to restore normal nasal function and appearance.<br>Procedure details: Rhinoplasty involves reshaping the nasal cartilage and bones to correct midline deformities, such as a split or flattened nasal bridge.<br>Categorization: Functional and Aesthetic — Improves breathing through the nose and enhances facial symmetry and appearance.<br>3. Forehead and Orbital Reconstruction (Craniofacial Surgery)<br>Procedure: For patients with frontonasal dysplasia or other cranial malformations, craniofacial reconstruction is often needed to reshape the forehead and correct orbital positioning (hypertelorism).<br>Procedure details: This surgery may involve frontal bone remodeling and orbital osteotomies to reposition the eye sockets and reduce the distance between them.<br>Categorization: Aesthetic and Functional — Enhances facial structure,		

										improves vision, and normalizes orbital positioning.<br>4. Orthognathic Surgery (Jaw Surgery)<br>Procedure: Micrognathia (small jaw) or misalignment of the jaw may require orthognathic surgery to correct the bite and improve jaw function.<br>Procedure details: Mandibular distraction osteogenesis or jaw repositioning surgery may be used to lengthen and align the jawbones, improving both chewing and facial aesthetics.<br>Categorization: Functional and Aesthetic — Corrects jaw alignment, improves chewing and speech, and restores facial balance.<br>5. Correction of Craniofacial Clefts<br>Procedure: In more severe cases of midline craniofacial clefts, multiple reconstructive surgeries are required to repair clefts that involve the forehead, nose, and upper lip.<br>Procedure details: Surgeons use bone grafts, skin flaps, or other soft tissue reconstruction techniques to close the clefts and restore normal anatomy.<br>Categorization: Functional and Aesthetic — Restores structural integrity of the face and improves both function and appearance.<br>6. Scar Revision<br>Procedure: After cleft repairs and other reconstructive surgeries, scar revision can be performed to minimize scarring and enhance the		

										cosmetic outcome.<br>Procedure details: Techniques like laser therapy or surgical excision can reduce scar visibility and improve the texture and color of the skin.<br>Categorization: Aesthetic — Focuses on improving the appearance of scars for better cosmetic outcomes.		
**Moebius Syndrome**	CFS	In	Clinical evaluation, genetic testing	*HOX, TUBB3, PLXND1, REV3L*	Impact: facial paralysis, limb abnormalities, requires multidisciplinary care	0.00000364	Generally normal with proper management	Supportive care, surgical correction of anomalies	Correction of craniofacial anomalies, limb abnormalities	Facial Reanimation Surgery (Smile Surgery): Functional and Aesthetic (to restore facial movement and ability to smile).<br>Eyelid Surgery (Lagophthalmos Correction): Functional (to improve eye closure and protect the corneas).<br>Cleft Palate Repair (If Present): Functional and Aesthetic (to improve speech, feeding, and facial appearance).<br>Limb Reconstruction (for Hand/Foot Abnormalities): Functional (to correct limb deformities and improve function).<br>Orthognathic Surgery (Jaw Surgery): Functional and Aesthetic (to correct jaw alignment, improve chewing, speech, and facial symmetry).<br>Scar Revision: Aesthetic (to minimize scarring after surgeries).	Functional	(174, 246-249)
**Mowat Wilson Syn.**	SYN	Bo	Clinical evaluation, genetic testing	*ZEB2(SIP1/ZFHX1B)*	Impact: developmental delays, multiple organ involvement	0.00001667	Generally normal with proper management	Supportive care, management of symptoms	Summary: Bone Defects: Yes, potentially involving craniofacial bones, particularly in syndromic cases with midline	Orbital Reconstruction: Functional and Aesthetic (to correct craniofacial defects involving the orbit).<br>Eyelid Surgery (Ptosis Correction): Functional and Aesthetic (to improve vision and eyelid	Mixed	(34, 109, 250)

									defects such as basal encephalocele or cleft palate. Cartilage Defects: Yes, possibly involving nasal and facial cartilage in cases with associated craniofacial anomalies. Plastic Surgery Involvement: Yes, primarily in the repair of midline defects, cleft lip/palate, and facial reconstruction in syndromic cases.	appearance). Scar Revision: Aesthetic (to minimize scarring from craniofacial surgery).		
**Moyamoya Disease**	PT	N	Clinical evaluation, imaging studies	*RNF213*	Impact: stroke-like episodes, requires surgical intervention	0.00001000	Reduced, variable depending on management of symptoms	Surgical revascularization, supportive care	Not typically involved	Indirect Revascularization (EDAS): Functional (to promote new blood vessel growth and improve long-term blood flow to the brain). Direct Revascularization (STA-MCA Bypass): Functional (to immediately restore blood flow and reduce stroke risk). Combined Revascularization: Functional (to ensure both immediate and long-term improvement in brain blood flow). Scalp and Skin Management Post-Surgery: Aesthetic and Functional (to ensure proper healing and prevent complications). Scar Revision: Aesthetic (to minimize scarring after cranial surgery).	Neither	(4, 251-253)
**Multiple Endocrine**	PT	N	childhood or	*RET*	variable depending on early detection	0.00002857		surgical removal of affected	not involved		Neither	(1, 57, 58, 60, 83, 254-256)

**Neoplasia Type 2 (MEN2)**			adolescence				glands, monitoring, genetic counseling				
**Multiple Sclerosis**	PT	N	Clinical evaluation, MRI	*PHOX2B*	Impact: physical disability, requires supportive care	0.00100000	Variable, reduced in severe cases	Immunomodulatory therapy, supportive care	Not typically involved	Facial Muscle Weakness: MS can cause muscle weakness or paralysis, including facial muscles. This can lead to facial asymmetry, but this is due to muscle involvement, not bone abnormalities.	Neither	(26)
**Musculocontractural Ehlers-Danlos syndrome (mcEDS)**	SYN	Bo	Clinical evaluation, genetic testing	*CHST14, DSE*	Impact: hypermobility, skin fragility, requires multidisciplinary care	0.00000100	Variable, depends on severity of symptoms	Supportive care, management of symptoms	Summary: Bone Defects: Yes, including craniosynostosis and skeletal dysmorphisms such as scoliosis. Cartilage Defects: Yes, including joint abnormalities (contractures and hypermobility) and potential involvement of ear and nasal cartilage. Plastic Surgery Involvement: Significant, particularly in craniofacial surgery, contracture release, skin management, and scar revision. Musculocontractural Ehlers-Danlos Syndrome is a complex disorder requiring a multidisciplinary approach, with plastic surgeons playing a crucial role in addressing the	Cleft Palate Repair: Functional and Aesthetic (to restore feeding, speech, and facial appearance). Craniofacial Reconstruction: Aesthetic and Functional (to correct facial deformities and improve function). Skin Repair and Scar Management: Aesthetic and Functional (to improve wound healing and reduce scarring). Contracture Release Surgery: Functional (to restore joint mobility and correct deformities). Orthognathic Surgery: Functional and Aesthetic (to correct jaw deformities and improve chewing and speech). Kyphoscoliosis Correction: Functional (to improve posture, spinal alignment, and mobility). Scar Revision and Wound Healing Management: Aesthetic (to minimize scarring and improve healing outcomes).	Aesthetic	(120, 257, 258)

									craniofacial, musculoskeletal, and skin-related challenges associated with the condition. Early intervention and tailored management strategies are essential to improving the quality of life for individuals with mcEDS.			
**Neurocutaneous Syndromes**	PT	In	Clinical evaluation, genetic testing	*NF1, NF2, TSC1, TSC2*	Impact: varies depending on specific syndrome	0.00012500	Varies widely depending on specific syndrome	Supportive care, management of symptoms	Summary: Bone and Cartilage Defects: Varies among syndromes, with notable skeletal involvement in NF1 and TSC, while VHL and Sturge-Weber generally do not involve direct bone or cartilage defects. Plastic Surgery Involvement: Significant in managing skin lesions, craniofacial abnormalities, and reconstructive needs across several neurocutaneous syndromes. Neurocutaneous syndromes are complex conditions requiring a multidisciplinary approach, with	Neurofibromatosis (NF1 & NF2): Excision of neurofibromas, debulking surgery, scar revision. Tuberous Sclerosis Complex (TSC): Laser therapy for facial angiofibromas, excision of skin lesions, scar revision. Sturge-Weber Syndrome (SWS): Laser treatment for port-wine stains, excision of skin lesions. Von Hippel-Lindau Disease (VHL): Excision of hemangioblastomas, scar revision. Ataxia-Telangiectasia (A-T): Laser treatment for telangiectasias. Incontinentia Pigmenti (IP): Excision of skin lesions, dental reconstruction. Linear Nevus Sebaceous Syndrome (LNSS): Excision of nevus sebaceous, facial reconstruction, scar revision	Aesthetic	(7, 47, 259, 260)

									plastic surgeons playing a crucial role in addressing the cosmetic and functional challenges associated with these disorders. Early intervention and tailored treatment strategies can significantly improve the quality of life for individuals with these syndromes.			
**Neurocutaneous melanosis**	PT	N	Clinical evaluation, imaging studies	*NRAS*	Impact: large pigmented moles, neurological issues, requires multidisciplinary care	0.00000100	Varies widely depending on specific syndrome	Supportive care, management of symptoms	Summary: Bone and Cartilage Defects: Varies among syndromes, with notable skeletal involvement in NF1 and TSC, while VHL and Sturge-Weber generally do not involve direct bone or cartilage defects. Plastic Surgery Involvement: Significant in managing skin lesions, craniofacial abnormalities, and reconstructive needs across several neurocutaneous syndromes. Neurocutaneous syndromes are complex conditions requiring a	Neurofibromatosis (NF1 & NF2): Excision of neurofibromas, debulking surgery, scar revision. Tuberous Sclerosis Complex (TSC): Laser therapy for facial angiofibromas, excision of skin lesions, scar revision. Sturge-Weber Syndrome (SWS): Laser treatment for port-wine stains, excision of skin lesions. Von Hippel-Lindau Disease (VHL): Excision of hemangioblastomas, scar revision. Ataxia-Telangiectasia (A-T): Laser treatment for telangiectasias. Incontinentia Pigmenti (IP): Excision of skin lesions, dental reconstruction. Linear Nevus Sebaceous Syndrome (LNSS): Excision of nevus sebaceous, facial reconstruction, scar revision	Aesthetic	(197-199)

									multidisciplinary approach, with plastic surgeons playing a crucial role in addressing the cosmetic and functional challenges associated with these disorders. Early intervention and tailored treatment strategies can significantly improve the quality of life for individuals with these syndromes.			
**Neuroblastoma**	PT	N	Clinical evaluation, biopsy	*PHOX2B, TRK/NT, ALK, KIF1B*	Impact: cancer, requires chemotherapy and supportive care	0.00014286	Reduced, variable depending on severity of symptoms	Surgical removal of nevi, supportive care	Removal of large nevi, correction of associated anomalies	Plastic and reconstructive surgery is not typically involved in the primary management of neuroblastoma, which is centered on oncologic tumor resection combined with chemotherapy and/or radiation therapy. However, PRS may be secondarily required following resection of large tumors to restore soft tissue coverage, abdominal wall integrity, or skeletal stability. Additional PRS involvement may address radiation-induced fibrosis, soft tissue deformities, or complex scarring, particularly in pediatric patients. In rare cases of osseous or limb involvement, reconstructive procedures may be necessary to preserve function. These interventions are supportive rather than central to neuroblastoma treatment.	Neither	(23, 24, 27, 28)

**Noonan syndrome**	SYN	Bo	Clinical evaluation, genetic testing	*PTPN11, KRAS, SOS1, RAF1, NRAS, BRAF, RIT1, SOS2, LZTR1, SPRED2Aortic arch arteries*	Impact: developmental delays, heart defects, requires multidisciplinary care	0.00057143	Generally normal with proper management	Supportive care, surgical correction of anomalies	Craniofacial Involvement:<br><br>Bone Defects: Craniofacial bone defects in Noonan syndrome contribute to the characteristic facial appearance, such as the broad forehead, down-slanting eyes, and low-set ears. These features are often subtle but distinctive.<br>Cartilage Defects:<br>The low-set, posteriorly rotated ears may involve abnormal development of the ear cartilage, contributing to their characteristic shape and position.<br>Plastic Surgery Correction:<br><br>Craniofacial Corrections:<br><br>Ptosis Repair: Surgery may be required to correct ptosis (drooping eyelids) if it interferes with vision.<br>Otoplasty: If the ear abnormalities are severe or cause significant cosmetic concerns,	Ptosis Correction (Eyelid Surgery):<br><br>Procedure: Corrects droopy eyelids (ptosis) that may affect vision.<br>Categorization: Functional and Aesthetic — Improves vision and enhances facial appearance.<br>Correction of Chest Deformities (Pectus Excavatum/Pectus Carinatum):<br><br>Procedure: Surgical correction of sunken or protruding chest deformities.<br>Categorization: Functional and Aesthetic — Improves breathing and chest appearance.<br>Rhinoplasty (Nasal Surgery):<br><br>Procedure: Reshapes the nose to correct wide nasal bridge or flat nasal appearance.<br>Categorization: Aesthetic — Improves facial balance and nasal shape.<br>Correction of Lymphedema:<br><br>Procedure: Surgical interventions or lymphatic drainage to reduce swelling in hands or feet due to lymphatic abnormalities.<br>Categorization: Functional — Reduces swelling and improves mobility.<br>Ear Reconstruction (Otoplasty):<br><br>Procedure: Reshapes and repositions low-set or rotated ears.<br>Categorization: Aesthetic	Aesthetic	(86, 88, 116, 213, 261-264)

									otoplasty (ear surgery) may be considered to reposition or reshape the ears.	— Enhances ear appearance and facial symmetry.<br>Scar Revision:<br><br>Procedure: Minimizes visible scarring after surgical interventions.<br>Categorization: Aesthetic — Improves the appearance of scars.<br>Functional Improvement: Enhances vision, breathing, and mobility through ptosis correction, chest deformity repair, and treatment for lymphedema.<br>Aesthetic Enhancement: Improves facial and ear appearance, reshapes the nose, and minimizes scarring.<br>These procedures aim to address the functional challenges and cosmetic concerns associated with Noonan syndrome, improving quality of life and physical appearance.		
**Oculocutaneous Albinism**	CO	N	Clinical evaluation, genetic testing	*TYR (OCA1A,OCA1B2), OCA2 (OCA2), TYRP1 (OCA3), SLC45A2 MATP(OCA4), SLC45A2 SLC24A5 (OCA6), LRMDA (OCA7), DCT (OCA8)(OCA4)*	Impact: vision problems, skin issues, requires supportive care	0.00005405	Generally normal with proper management	Supportive care, management of symptoms	Not typically involved	While plastic surgery is not commonly required for OCA, dermatological care and eye management are crucial for maintaining skin health and improving visual function.<br><br>1. Skin Care and Sun Protection<br>Procedure: Due to the lack of melanin, individuals with OCA are at high risk of skin damage and skin cancers from UV exposure. Comprehensive sun protection measures are critical.<br>Procedure details: Regular use of broad-spectrum sunscreen, protective clothing, hats, and UV-	Neither	(265, 266)

										blocking sunglasses is recommended to minimize the risk of skin cancer.<br>Categorization: Functional and Preventative — Reduces the risk of skin damage and skin cancer.<br>2. Scar Revision (If Needed for Skin Cancer Excision)<br>Procedure: Individuals with OCA are prone to developing basal cell carcinoma, squamous cell carcinoma, and other types of skin cancer. After excision, scar revision surgery may be necessary.<br>Procedure details: After the surgical removal of skin cancers, techniques such as scar revision or laser therapy can be used to improve the cosmetic appearance of the scar.<br>Categorization: Aesthetic — Improves the appearance of scars following cancer treatment.<br>3. Eye Surgery (Strabismus Surgery)<br>Procedure: Many individuals with OCA experience strabismus (misalignment of the eyes). Strabismus surgery can help correct the alignment and improve vision.<br>Procedure details: Extraocular muscle surgery is performed to reposition the muscles that control eye movement, allowing the eyes to align correctly.<br>Categorization: Functional and Aesthetic — Improves vision by correcting eye alignment, and enhances facial appearance.<br>4. Vision Correction (Optical Management)		

										Procedure: Individuals with OCA often require glasses or contact lenses to correct refractive errors (e.g., myopia, hyperopia, or astigmatism) and improve visual acuity. Procedure details: Tinted lenses may also be used to reduce photophobia (light sensitivity). Categorization: Functional — Improves vision and reduces light sensitivity. 5. Cosmetic Procedures for Hair and Skin Procedure: Some individuals with OCA may opt for cosmetic procedures to address aesthetic concerns, such as hair coloring or makeup to enhance skin pigmentation. Procedure details: Hair dyes and tinted skin products can be used to achieve a desired appearance. Categorization: Aesthetic — Enhances appearance and allows for more personalized cosmetic choices.		
**Oculocerebrocutaneous (Delleman) Syndrome**	CFS	In	Clinical evaluation, imaging studies	-	Impact: vision problems, neurological issues, requires multidisciplinary care	0.00000100	Reduced, variable depending on severity of symptoms	Supportive care, surgical correction of anomalies	Correction of craniofacial anomalies	Reconstruction of Ocular Structures (for Colobomas, Anophthalmia, or Microphthalmia): Procedure: Reconstructive surgery or prosthetics to address underdeveloped or missing eyes. Categorization: Functional and Aesthetic — Improves vision (if possible) and facial appearance. Facial Reconstruction (for Facial Asymmetry or Skin Tags): Procedure: Removal of	Aesthetic	(267, 268)

										skin tags or lipomas on the face, often requiring soft tissue reconstruction to maintain symmetry.<br>Categorization: Aesthetic — Enhances facial appearance by addressing asymmetry and skin anomalies.<br>Ear Reconstruction (Otoplasty):<br><br>Procedure: Surgical reshaping of malformed ears, particularly if there are ear tags or deformities.<br>Categorization: Aesthetic — Improves the appearance and positioning of the ears.		
**Orofaciodigital Syndrome I**	SYN	Bo	Clinical evaluation, genetic testing	*OFD1, C2CD3, KIAA0753, SCLT1, TBC1D32, DDX59, INTU, CPLANE1, TCTN3, TMEM107, TMEM231, TMEM138, IFT157CXORF5*	Impact: craniofacial and digital abnormalities, requires surgical correction	0.00000667	Generally normal with proper management	Surgical correction of craniofacial and digital anomalies	Correction of craniofacial and digital anomalies	Cleft Lip and Palate Repair:<br><br>Procedure: Surgical repair of the cleft lip and/or palate to restore normal oral function.<br>Categorization: Functional and Aesthetic — Restores feeding, speech, and facial appearance.<br>Syndactyly or Polydactyly Correction:<br><br>Procedure: Removal of extra digits (polydactyly) or separation of webbed digits (syndactyly) to improve hand or foot function.<br>Categorization: Functional and Aesthetic — Restores normal hand or foot function and appearance.<br>Facial Reconstruction:<br><br>Procedure: Address facial dysmorphism, such as jaw malformations or nasal deformities.<br>Categorization: Aesthetic	Mixed	(90, 269)

										and Functional — Improves facial structure and function, particularly in cases of jaw abnormalities.<br>Dental Reconstruction:<br><br>Procedure: Corrective dental surgery for missing or misshapen teeth.<br>Categorization: Functional and Aesthetic — Restores normal chewing and speaking ability while improving dental appearance.		
**Paragangliomas**	PT	N	Imaging studies (CT, MRI, ultrasound)	*SDHB, SDHC, SDHD, SDHAF2, SDHA, SLC25A11, DLST*	Impact: depends on location and size, requires surgical removal	0.00002500	Variable, depends on size and location of tumors	Surgical removal of paragangliomas	Craniofacial Involvement:<br><br>Bone Defects: Paragangliomas do not directly cause bone defects, but those in the head and neck region may cause bone erosion or displacement due to their growth.<br><br>Cartilage Defects: No specific cartilage defects are associated with paragangliomas, but the tumors may affect nearby structures, including the larynx or ear.<br><br>Plastic Surgery Correction:<br><br>Craniofacial Corrections: Plastic surgery may be involved	1. Tumor Excision<br>Procedure: Surgical excision of paragangliomas is the preferred treatment, particularly for head and neck tumors or those causing mass effects or secreting hormones.<br>Procedure details: Depending on the tumor location, this surgery can be highly complex, especially if the tumor is near vital structures like nerves or blood vessels (e.g., carotid artery). Surgeons may collaborate with vascular surgeons, neurosurgeons, or ENT specialists.<br>Categorization: Functional — Removes the tumor to alleviate symptoms and prevent complications such as metastasis or local tissue invasion.<br>2. Reconstruction After Tumor Excision (Head and Neck)<br>Procedure: Reconstructive surgery is often necessary after the removal of paragangliomas in the head and neck region, particularly if there are	Neither	(110, 270, 271)

									post-resection to address defects in soft tissue or bone caused by tumor removal.	large defects or if important structures like the jaw, ear, or soft tissue are affected.<br>Procedure details: Reconstructive techniques may involve flap surgery, bone grafting, or soft tissue reconstruction to restore function and appearance.<br>Categorization: Functional and Aesthetic — Restores facial symmetry, jaw function, and tissue integrity after tumor removal.<br>3. Vascular Reconstruction (If Required)<br>Procedure: Some paragangliomas, particularly those located near large blood vessels (e.g., carotid body tumors), may require vascular reconstruction to maintain blood flow after tumor removal.<br>Procedure details: Vascular surgeons may need to repair or reconstruct major arteries or veins after tumor excision.<br>Categorization: Functional — Restores or preserves blood flow to critical regions of the body, particularly in the head and neck.<br>4. Scar Revision Surgery<br>Procedure: After paraganglioma removal in visible areas, patients may undergo scar revision to minimize the cosmetic impact of the surgery.<br>Procedure details: Techniques like laser therapy, dermabrasion, or scar excision may be used to reduce scarring.<br>Categorization: Aesthetic — Improves cosmetic		

										outcomes by minimizing scar visibility.		
**PCWH, Waardenburg predominance (Peripheral demyelinating neuropathy, central dysmyelinating leukodystrophy, Waardenburg syndrome and Hirschsprung disease)**	SYN	In		*PAX3 (WS1,3). MITF (WS2A) SNAI2 (WS2D), EDNRB/EDN3, SOX10 (WS4), ACTG1*		0.00000100			Craniofacial Involvement:<br><br>Bone Defects: Craniofacial bone defects are not a primary feature of PCWH syndrome. However, craniofacial asymmetries and the structural anomalies seen in Waardenburg syndrome (e.g., dystopia canthorum, broad nasal bridge) are related to underlying bone structure.<br>Cartilage Defects: Cartilage defects are not typically a hallmark of PCWH syndrome, but structural anomalies related to facial cartilage (e.g., nasal cartilage) may be present in the context of Waardenburg syndrome.<br>Plastic Surgery Correction:<br><br>Craniofacial Corrections:<br><br>Facial Reconstruction: In cases where facial asymmetries or	Facial Reconstruction (for Dysmorphism):<br><br>Procedure: Correction of a wide nasal bridge or other facial asymmetries.<br>Categorization: Aesthetic — Improves facial appearance by addressing structural abnormalities.<br>Heterochromia Management (If Desired):<br><br>Procedure: Cosmetic procedures, such as contact lenses, to address eye color differences if they are a cosmetic concern.<br>Categorization: Aesthetic — Enhances the cosmetic appearance of the eyes.<br>Hearing Aids or Cochlear Implants:<br><br>Procedure: For sensorineural hearing loss, cochlear implants or hearing aids are often recommended.<br>Categorization: Functional — Improves hearing ability.<br>Correction of Pigmentation Abnormalities:<br><br>Procedure: Cosmetic options, such as laser therapy, for large areas of hypopigmentation or vitiligo-like patches.<br>Categorization: Aesthetic — Restores a more uniform skin tone and appearance.<br>Surgery for Hirschsprung Disease:<br><br>Procedure: Surgical removal of the affected	Neither	(21, 37, 39, 41, 43-45, 272)

									structural anomalies are pronounced, plastic surgery might be considered to correct these defects, particularly for functional or aesthetic reasons. Correction of Nasal Deformities: Surgical intervention may be needed to address nasal structure abnormalities associated with Waardenburg syndrome. Other Corrections:<br><br>While plastic surgery is not typically involved in treating the neurological aspects of the syndrome, it may play a role in managing visible craniofacial defects and improving the quality of life for affected individuals.	portion of the colon in cases of Hirschsprung disease. Categorization: Functional — Restores normal bowel function and relieves intestinal obstruction.		
**Peter's Anomaly**	CO	N	Clinical evaluation, imaging studies	*PAX6, PITX2, FOXE3, FOXC1, MAF, PITX3, CYP1B1*	Impact: vision loss, requires surgical correction	0.00000667	0	Generally normal with proper management	Surgical correction of ocular anomalies	Correction of ocular anomalies	Neither	(273-277)
**PHACE (Posterior fossa**	SYN	In	Clinical evaluatio	*Various genetic and*	Impact: multiple organ	0.00002667	Generally normal	Surgical correction of	Supportive care, surgical	PHACE Syndrome Craniofacial Involvement:	Aesthetic	(4, 245, 259, 260, 278)

**malformations, Hemangiomas (large vascular birthmarks on the face), Arterial anomalies, Cardiac defects, Eye abnormalities, and Sternal Breast Bone Defects)**			n, imaging studies	*environmental factors*	involvement, requires multidisciplinary care		with proper management	ocular anomalies	correction of anomalies	Bone Defects: Craniofacial bone anomalies may include skull and jaw malformations, particularly if associated with a posterior fossa brain malformation. Cartilage Defects: Cartilage defects are not a primary feature of PHACE syndrome, but vascular anomalies could potentially affect facial cartilage development. Plastic Surgery Correction: Plastic surgery may be necessary for hemangiomas, particularly large ones that cause facial disfigurement, as well as for reconstructive procedures related to craniofacial abnormalities		
**Pheochromocytomas**	PT	N	Imaging studies (CT, MRI, MIBG scan)	*VHL, RET, SDHB, SDHD, TMEM127, MAX*	Impact: hypertension, requires surgical removal of tumors	0.00008000	Generally normal with proper management	Dietary management, supportive care	Not typically involved	Plastic and reconstructive surgery is not typically involved in the primary treatment of pheochromocytomas, which are managed primarily by adrenalectomy. However, PRS may be indirectly required in select cases, particularly for reconstruction following large tumor resections, abdominal wall defects, or secondary complications such as hernias. In syndromic conditions associated with pheochromocytomas (e.g., MEN2 or neurofibromatosis type 1), PRS may also address functional reconstruction or aesthetic correction of associated lesions. These interventions are considered secondary and are not central to the	Neither	(21, 60, 62, 270, 271, 279-281)

									management of pheochromocytomas.		
**Piebaldism**	CO	N	Clinical evaluation, genetic testing	*KIT*	Impact: skin pigmentation issues, requires supportive care	0.00000080	Generally normal with proper management	Supportive care, management of symptoms	Piebaldism Craniofacial Involvement: Bone Defects: Piebaldism does not involve craniofacial bone defects. Cartilage Defects: No cartilage defects are associated with piebaldism. Plastic Surgery Correction: Plastic surgery may be considered for cosmetic reasons, such as skin grafting to repigment affected areas, though this is typically more related to dermatological treatment.'	1. Cleft Lip and Palate Repair Procedure: If a child is born with a cleft lip and/or palate, surgical repair is essential to restore normal feeding, speech, and facial appearance. Procedure details: Cheiloplasty (lip repair) and palatoplasty (palate repair) are performed to close the gaps in the lip and palate and improve oral function. Categorization: Functional and Aesthetic — Improves feeding, speech, and facial appearance. 2. Facial Reconstruction (for Facial Asymmetry or Midfacial Hypoplasia) Procedure: Facial reconstruction may be required for individuals with significant facial asymmetry or midfacial hypoplasia (underdevelopment of the midface). This could involve bone grafting, orthognathic surgery, or soft tissue reconstruction. Procedure details: Bone grafting or implants can be used to restore balance and symmetry to the facial structure, particularly in the cheekbones and jaw. Categorization: Aesthetic — Enhances facial appearance and symmetry. 3. Ear Reconstruction (for Microtia or Malformed Ears) Procedure: In cases of microtia (small or underdeveloped ears), ear reconstruction or otoplasty	Neither	(282, 283)

										can restore a more normal appearance and, in some cases, improve hearing.<br>Procedure details: Surgeons may use cartilage grafts (often from the ribs) to rebuild the external ear, creating a natural-looking ear shape.<br>Categorization: Aesthetic and Functional — Improves ear appearance and hearing function.<br>4. Rhinoplasty (for Nasal Abnormalities)<br>Procedure: Some individuals with piebaldism and craniofacial anomalies may have nasal abnormalities (e.g., a broad nasal bridge or other malformations). Rhinoplasty can be performed to correct these abnormalities and improve both function and appearance.<br>Procedure details: Surgeons reshape the nasal bones and cartilage to enhance the nose's structure and function.<br>Categorization: Aesthetic and Functional — Improves nasal function and facial aesthetics.<br>5. Skin Grafting or Melanocyte Transplantation (for Depigmented Skin)<br>Procedure: As in classic piebaldism, some patients may seek skin grafting or melanocyte transplantation to repigment the depigmented patches of skin.<br>Procedure details: Pigmented skin from another part of the body is grafted onto the depigmented areas, or		

										melanocyte transplantation is used to restore color. Categorization: Aesthetic — Restores skin pigmentation to improve cosmetic appearance.		
**Pierre Robin sequence**	SYN	Bo	Clinical evaluation, genetic testing	*SOX9, MED13L*	Impact: feeding and breathing difficulties, requires surgical correction	0.00005000	Generally normal with proper management	Surgical correction of craniofacial anomalies	Correction of craniofacial anomalies	1. Mandibular Distraction Osteogenesis (MDO) Procedure: Mandibular distraction osteogenesis is a surgical procedure used to lengthen the jawbone (mandible). This helps relieve airway obstruction by pulling the jaw forward and creating more space for the tongue and airway. Procedure details: The surgeon makes a cut in the mandible and uses a distraction device to gradually lengthen the bone over time. This helps correct the micrognathia and reduces glossoptosis. Categorization: Functional — Improves breathing and reduces airway obstruction. 2. Tongue-Lip Adhesion Procedure: For infants with severe airway obstruction, tongue-lip adhesion surgery is performed to temporarily attach the tongue to the lower lip, pulling it forward and preventing it from obstructing the airway. Procedure details: This surgery is usually performed early in life and may be reversed later as the child grows and the jaw develops. Categorization: Functional — Temporarily relieves airway obstruction caused by glossoptosis. 3. Cleft Palate Repair (Palatoplasty)	Functional	(112, 284-289)

										Procedure: Cleft palate repair is a key surgical procedure in PRS, as many children have an associated cleft palate. The goal of palatoplasty is to close the gap in the roof of the mouth, restoring normal function.<br>Procedure details: The cleft is surgically closed using tissue from either side of the gap. This procedure improves feeding, speech, and hearing function by restoring the palate.<br>Categorization: Functional and Aesthetic — Improves feeding, speech, and facial appearance.<br>4. Tracheostomy (for Severe Airway Obstruction)<br>Procedure: In severe cases of airway obstruction that cannot be resolved by mandibular distraction or tongue-lip adhesion, a tracheostomy may be necessary to create a direct airway through the neck.<br>Procedure details: A small opening is created in the windpipe (trachea) to bypass the obstructed upper airway.<br>Categorization: Functional — Provides an alternative airway to relieve breathing difficulties.<br>5. Orthognathic Surgery (for Older Children)<br>Procedure: As children with PRS grow, some may need orthognathic surgery to further correct jaw deformities and improve facial structure.<br>Procedure details: This procedure involves repositioning the upper and		

										lower jaws to improve the bite, jaw alignment, and overall facial appearance. Categorization: Functional and Aesthetic — Improves jaw function, chewing, and facial aesthetics. 6. Scar Revision (Following Cleft Palate Repair) Procedure: After cleft palate repair or other surgeries, some patients may have visible scars. Scar revision surgery can be performed to minimize scarring and improve the aesthetic outcome. Procedure details: Techniques such as laser therapy or surgical scar revision may be used to improve scar appearance. Categorization: Aesthetic — Focuses on reducing scarring for a better cosmetic result		
**Postaxial Acrofacial Dysostosis Syndrome (Miller Syndrome)**	SYN	Bo	Clinical evaluation, genetic testing	*DHODH*	Impact: craniofacial and limb abnormalities, requires surgical correction	0.00009091	Generally normal with proper management	Surgical correction of craniofacial and limb anomalies	Correction of craniofacial and limb anomalies	1. Cleft Lip and Palate Repair Procedure: Cleft lip and/or palate repair is typically performed in early childhood to restore normal function for feeding, speech, and breathing. Procedure details: Palatoplasty and cheiloplasty are performed to close the gap in the lip and/or palate, restoring normal oral function and facial appearance. Categorization: Functional and Aesthetic — Improves feeding, speech, and facial appearance. 2. Mandibular Reconstruction (Jaw Surgery) Procedure: Patients with	Functional	(186, 290)

										micrognathia (small jaw) may require mandibular reconstruction to correct jaw underdevelopment. This surgery helps improve both breathing and appearance.<br>Procedure details: Techniques such as distraction osteogenesis (gradual lengthening of the jaw) or bone grafting can be used to enhance jaw size and function.<br>Categorization: Functional and Aesthetic — Corrects jaw underdevelopment, improving breathing and facial symmetry.<br>3. Orbital and Zygomatic Reconstruction (Cheekbone Reconstruction)<br>Procedure: Patients with underdeveloped cheekbones (zygomatic bones) may need orbital and zygomatic reconstruction to enhance facial structure.<br>Procedure details: Bone grafting or implants may be used to restore cheekbone contour and provide facial support.<br>Categorization: Aesthetic — Improves facial symmetry and appearance.<br>4. Limb Reconstruction or Pollicization (Thumb Reconstruction)<br>Procedure: Limb abnormalities, such as missing or underdeveloped fingers or toes, may require limb reconstruction. Pollicization is the creation of a functional thumb from an existing finger, a common procedure for postaxial limb defects.		

										Procedure details: Reconstructive hand surgery may involve repositioning or reshaping existing digits, and pollicization provides thumb functionality if the thumb is absent.<br>Categorization: Functional — Restores hand function and dexterity.<br>5. Ear Reconstruction (Otoplasty)<br>Procedure: Otoplasty is performed to reconstruct low-set or malformed ears, improving both appearance and hearing function when combined with hearing aids or bone-anchored hearing devices (BAHA) for those with hearing loss.<br>Procedure details: Surgeons may use cartilage grafts to reconstruct the external ear and create a more typical ear shape.<br>Categorization: Functional and Aesthetic — Improves hearing function and ear appearance.<br>6. Scar Revision (After Facial Surgery)<br>Procedure: Following surgeries for craniofacial corrections, patients may develop noticeable scars. Scar revision can be performed to improve the cosmetic outcomes.<br>Procedure details: Techniques such as laser therapy or dermabrasion can reduce scar visibility.<br>Categorization: Aesthetic — Enhances cosmetic results by minimizing scarring.<br>7. Orthognathic Surgery (Jaw Alignment in Adolescence)<br>Procedure: As patients		

										grow, they may require additional orthognathic surgery to further align the upper and lower jaws for improved bite and facial symmetry.<br>Procedure details: This surgery involves repositioning the upper and/or lower jaws to correct misalignment and improve function.<br>Categorization: Functional and Aesthetic — Corrects jaw alignment, improves chewing, and enhances facial balance.<br><br>Key Surgical Goals:<br>Functional Improvement: Focused on improving feeding, speech, breathing, and hand functionality through cleft palate repair, jaw surgery, and limb reconstruction.<br>Aesthetic Enhancement: Procedures such as cheekbone reconstruction, ear reshaping, and scar revision aim to improve facial symmetry and overall appearance.		
**SAMS (Short Stature, Auditory Canal Atresia, Mandibular Hypoplasia, and Skeletal Abnormalities) Syndrome**	SYN	Bn	Clinical evaluation, genetic testing	*GSC*	Impact: craniofacial and skeletal abnormalities, requires surgical correction	0.00000100	Generally normal with proper management	Surgical correction of craniofacial and skeletal anomalies	Correction of craniofacial and skeletal anomalies	1. Facial Reconstruction (for Craniofacial Abnormalities)<br>Procedure: Facial reconstruction may be necessary for individuals with craniofacial dysmorphisms, such as micrognathia or other facial asymmetries. This surgery aims to correct facial structure and improve both function (e.g., breathing, chewing) and aesthetics.<br>Procedure details: Techniques such as bone grafting or distraction	Mixed	(256, 291, 292)

										osteogenesis (gradual bone lengthening) may be used to correct jaw or facial abnormalities. Categorization: Functional and Aesthetic — Improves facial symmetry, jaw function, and overall appearance. 2. Skin Grafting (for Skin Defects) Procedure: For individuals with significant skin defects such as areas of skin atrophy or severe pigmentation abnormalities, skin grafting may be performed to restore the affected areas. Procedure details: Split-thickness or full-thickness skin grafts are used to replace damaged or missing skin, improving both function and appearance. Categorization: Aesthetic and Functional — Restores skin integrity and improves the cosmetic appearance of the affected areas. 3. Limb Reconstruction (for Bone Abnormalities) Procedure: Patients with abnormal limb development or skeletal deformities may require limb reconstruction to correct malformations and restore function. Procedure details: This can include osteotomies (bone cutting and repositioning), bone lengthening, or the use of orthopedic devices to correct misaligned bones and improve mobility. Categorization: Functional — Restores limb function and mobility.		

										4. Scoliosis Surgery<br>Procedure: For patients with severe scoliosis (curvature of the spine), spinal fusion surgery or other corrective spinal procedures may be necessary to prevent further progression of the curvature and improve posture.<br>Procedure details: Spinal fusion involves fusing the affected vertebrae together using bone grafts, rods, and screws to stabilize the spine.<br>Categorization: Functional — Corrects spinal deformities, improving posture and reducing the risk of further complications.<br>5. Jaw Surgery (Orthognathic Surgery)<br>Procedure: For patients with micrognathia (small jaw), orthognathic surgery may be performed to realign the upper and lower jaws, improving both facial structure and function (e.g., breathing and chewing).<br>Procedure details: This involves repositioning the jawbones through a surgical procedure, often combined with bone grafting or distraction osteogenesis.<br>Categorization: Functional and Aesthetic — Improves jaw function and facial appearance.<br>6. Scar Revision Surgery<br>Procedure: Patients with visible scars resulting from surgical interventions or skin defects may benefit from scar revision to improve the appearance of		

										these scars. Procedure details: Techniques such as laser therapy, surgical scar excision, or dermabrasion can help minimize scar visibility. Categorization: Aesthetic — Enhances cosmetic outcomes by reducing scarring.		
**Seckel Syndrome / Microcephalic Osteodyplastic Primordial Dwarfism, Type II (MOPD2)**	SYN	Bo	Typically diagnosed at birth or in early infancy due to noticeable growth retardation, microcephaly, and distinctive facial features.	*Mutations in the ATR, RBBP8, CENPJ, CEP152, CEP63, NIN, DNA2, TRAIP, NSMCE2, and CEP295* genes are associated with Seckel Syndrome, impacting DNA repair pathways and cell cycle regulation. *MOPD2 Similar but also distinct phenotypes to Seckel Syndrome caused by Mutations in Pericentrin (See OMIM #210720)	Life Expectancy: Individuals with Seckel Syndrome often live into adulthood, though life expectancy may be reduced depending on the severity of symptoms. Quality of Life: Varies depending on the degree of intellectual disability and physical symptoms. Individuals may experience developmental delays and require ongoing support for daily living.	0.00000100				1. Mandibular Reconstruction (Jaw Surgery) Procedure: Mandibular reconstruction is often necessary for individuals with micrognathia (small jaw) to improve breathing and feeding functions, as well as facial symmetry. Procedure details: Techniques such as distraction osteogenesis (gradual lengthening of the jawbone) or bone grafting may be used to correct the small, underdeveloped jaw. Categorization: Functional and Aesthetic — Improves jaw function, breathing, and facial appearance. 2. Correction of Craniofacial Abnormalities Procedure: Patients with Seckel Syndrome often have facial asymmetry or abnormal cranial shapes. Reconstructive surgery may be needed to correct these abnormalities. Procedure details: Cranial vault remodeling or orbital surgery may be performed to address abnormalities of the skull or eye sockets. Categorization: Aesthetic and Functional — Enhances facial appearance and may improve vision or	Functional	(293-296)

										breathing, depending on the nature of the craniofacial abnormalities.<br>3. Rhinoplasty (Nasal Surgery)<br>Procedure: Some individuals with Seckel Syndrome have a prominent, beak-like nose. Rhinoplasty can be performed to reshape the nose and improve facial proportions.<br>Procedure details: Nasal reshaping may involve the use of cartilage grafts or soft tissue adjustments to reduce the prominence of the nose.<br>Categorization: Aesthetic — Focuses on improving facial symmetry and the cosmetic appearance of the nose.<br>4. Cleft Palate Repair (If Present)<br>Procedure: In rare cases, individuals with Seckel Syndrome may have a cleft palate, requiring palatoplasty to restore normal feeding and speech functions.<br>Procedure details: Cleft palate repair involves closing the gap in the roof of the mouth to restore the separation between the nasal and oral cavities.<br>Categorization: Functional and Aesthetic — Improves feeding, speech, and facial appearance.<br>5. Orthognathic Surgery (Jaw Realignment)<br>Procedure: For older children or adults with significant jaw misalignment, orthognathic surgery may be needed to reposition the upper and lower jaws, improving		

										chewing and facial balance.<br>Procedure details: The procedure involves repositioning the jawbones and may be combined with other reconstructive techniques.<br>Categorization: Functional and Aesthetic — Improves bite, chewing, and facial appearance.<br>6. Correction of Skeletal Deformities<br>Procedure: In cases where patients develop scoliosis or other skeletal deformities, orthopedic or plastic surgeons may collaborate to correct spinal or limb deformities.<br>Procedure details: This may involve spinal fusion surgery for scoliosis or limb reconstruction for other skeletal abnormalities.<br>Categorization: Functional — Improves mobility and prevents further skeletal complications.<br><br>Functional Improvement: Surgery aims to improve essential functions like breathing, chewing, and feeding through jaw reconstruction, cleft palate repair, and skeletal correction.<br>Aesthetic Enhancement: Procedures such as nasal surgery, cranial remodeling, and scar revision focus on improving the appearance of the face and correcting craniofacial abnormalities.		
**Sensorineural Hearing Impairment**	CO	N	Clinical evaluatio n,	*Various genes (GREB1L,*		0.00200 000				While plastic surgery is generally not involved in the direct treatment of	Neither	(121)

			audiometric testing	*MASP1, SIX1, TAF1)*						SNHI, certain surgical interventions related to hearing restoration or craniofacial syndromes that involve hearing loss can be relevant.		
**Short-Rib Thoracic Dysplasia**	PT	In	Clinical evaluation, imaging studies	*IFT172, IFT80, IFT81, IFT140, IFT152, IFT43, DYNC2H1, TTC21B, WDR19, NEK1, WDR35, WDR60, WDR34, CEP120, KIAA0586, DYNC2LI1, TCTEX1D2, INTU, KIAA0753, FGF4*		0.00000100			Correction of limb anomalies	While plastic surgery may not directly address the core thoracic issues, reconstructive and orthopedic surgeries can be essential in managing the skeletal abnormalities and respiratory complications that arise from the condition. Here are some relevant surgical interventions for SRTD10:<br><br>1. Thoracic Expansion Surgery (VEPTR - Vertical Expandable Prosthetic Titanium Rib)<br>Procedure: In severe cases of thoracic hypoplasia, where the chest cavity is too small to allow normal lung development, thoracic expansion surgery can be performed. The VEPTR (Vertical Expandable Prosthetic Titanium Rib) procedure involves the implantation of expandable rods along the ribs and spine to increase the size of the chest cavity and improve lung function.<br>Procedure details: Expandable devices are surgically placed and adjusted over time to expand the chest wall as the child grows, allowing for better lung expansion and respiratory function.<br>Categorization: Functional — Improves respiratory capacity by expanding the chest cavity, which is critical for survival and	Neither	(89, 91, 92, 297)

										quality of life.<br>2. Limb Lengthening Surgery (for Shortened Long Bones)<br>Procedure: Some individuals with SRTD10 may have shortened limbs due to skeletal dysplasia. In such cases, limb lengthening surgery can be performed to increase the length of the bones in the arms and legs, improving mobility and function.<br>Procedure details: This is usually done through distraction osteogenesis, where the bone is surgically cut and a device is used to gradually lengthen the bone as it heals.<br>Categorization: Functional and Aesthetic — Improves mobility and limb function while also enhancing the appearance of the limbs.<br>3. Polydactyly Correction (for Extra Digits)<br>Procedure: If polydactyly (extra fingers or toes) is present, surgical removal of the extra digits may be considered to improve hand or foot function and appearance.<br>Procedure details: The extra digit is surgically removed, and the hand or foot is reconstructed to restore a more typical appearance and functionality.<br>Categorization: Functional and Aesthetic — Improves hand or foot function and restores a more normal appearance.<br>4. Orthopedic Surgery for Skeletal Abnormalities (Spine and Pelvis)<br>Procedure: Some		

										individuals with SRTD10 develop scoliosis or other deformities in the spine or pelvis. Orthopedic surgery may be necessary to correct these deformities and improve posture, mobility, and overall quality of life. Procedure details: This may involve spinal fusion or the use of bracing to stabilize the spine and pelvis. Categorization: Functional — Improves posture, mobility, and reduces pain from skeletal deformities.		
**Split-hand/split-foot malformation 4**	PT	Bn	Clinical evaluation, genetic testing	*TP63, DLX5, WNT10B*	Impact: limb abnormalities, requires surgical correction	0.00001053			Correction of limb anomalies	Rrare genetic condition that affects the development of the central rays of the hands and feet, leading to a split or cleft appearance. It is caused by mutations in the TP63 gene and can range in severity, from mild webbing or missing digits to more severe deformities with missing bones (phalanges, metacarpals, metatarsals). Affected individuals may also have other symptoms, including intellectual impairment, ectodermal anomalies, and orofacial clefts.	Functional	(63, 64, 98, 99, 101)
**SUPT16H-associated neurodevelopmental disorder and neurocristopathy**	SYN	Bn	Infancy/childhood due to early neurodevelopmental findings	*SUPT16H*	Variable and often chronically affected by developmental and neurologic needs.	0.000001	Ultra rare	Supportive and symptom-directed (neurologic follow-up, seizure management when needed, craniofacial hearing evaluation	Correction of craniofacial and/or ear anomalies when present	No syndrome-specific procedures were reported that apply to all patients. If ear malformations are present, procedures may include auricular reconstruction or prosthetic ear management; if aural atresia is present, surgical repair may be considered	Mixed	(298)

								when relevant				
**Tietz Syndrome**	PT	C	Clinical evaluation, imaging studies	*MITF*	Impact: chest pain, requires supportive care	0.00001000	Generally normal with proper management	Surgical correction of limb anomalies	Tietz syndrome is a congenital condition marked by profound sensorineural hearing loss, light pigmentation of the hair and skin, and distinctive ocular features. At birth, individuals present with white hair and very pale skin, although hair may darken to blond or red over time. The skin is photosensitive, with potential for mild tanning or freckling upon sun exposure. Eye findings include blue irides and reduced pigmentation in the retinal pigment epithelium, which may be evident on eye exam, though the impact on vision is uncertain.	There is no mention of any plastic surgery intervention in the literature reviewed, including the case study by Izumi et al. The phenotype does not involve structural craniofacial anomalies typically requiring surgical correction, and management appears to be primarily supportive and audiologic.	Neither	(118, 299)
**Treacher Collins Syndrome**	SYN	Bo	Clinical evaluation, genetic testing	*TCOF1, POLR1B. POLR1C, POLR1D*	Impact: craniofacial abnormalities, requires surgical correction	0.00002000	Generally normal with proper management	Supportive care, pain management	Treacher Collins syndrome (TCS) is a genetic disorder characterized by craniofacial deformities, including underdeveloped facial bones, cleft palate, and ear	1. Mandibular Reconstruction (Jaw Surgery)<br>Procedure: Mandibular distraction osteogenesis or reconstructive jaw surgery is performed to lengthen the underdeveloped mandible, improving airway function and facial balance.	Functional	(6, 125, 300-303)

									abnormalities. The syndrome is caused by mutations in the TCOF1, POLR1C, or POLR1D genes.<br><br>Craniofacial Involvement:<br><br>Bone Defects: Craniofacial bone defects are a hallmark of TCS, including hypoplasia of the zygomatic arches, mandibular hypoplasia (small jaw), and underdeveloped or absent cheekbones.<br>Cartilage Defects: Ear anomalies, including microtia (small or underdeveloped ears), involve cartilage defects. The nasal cartilage may also be affected, leading to breathing difficulties.<br>Plastic Surgery Correction: Extensive plastic and reconstructive surgery is often required to correct facial asymmetry, jaw defects, and ear reconstruction.<br>Cleft palate	Procedure details: The surgeon gradually lengthens the jawbone using a distraction device over time to correct micrognathia.<br>Categorization: Functional and Aesthetic — Improves breathing, feeding, and facial appearance.<br>2. Cleft Palate Repair<br>Procedure: Palatoplasty is performed to close the gap in the roof of the mouth, restoring normal oral function for feeding and speech.<br>Procedure details: Tissue is used from either side of the cleft to reconstruct the palate, enabling proper speech development and swallowing.<br>Categorization: Functional and Aesthetic — Improves speech and feeding while enhancing facial appearance.<br>3. Ear Reconstruction (Otoplasty or Microtia Repair)<br>Procedure: Reconstructive surgery to rebuild external ears (microtia repair) or improve the appearance of small or absent ears.<br>Procedure details: Surgeons use cartilage grafts (often from the ribs) to reconstruct the external ear, and in some cases, prosthetic ears are considered.<br>Categorization: Functional and Aesthetic — Improves hearing with prosthetic devices and restores ear appearance.<br>4. Zygomatic Reconstruction (Cheekbone Reconstruction)		

									repair and mandibular advancement surgeries are common. Multiple surgeries are usually needed over time.	Procedure: Zygomatic reconstruction is performed to address underdeveloped cheekbones, providing better midface support and symmetry. Procedure details: Bone grafts or implants are used to enhance cheekbone structure and improve facial balance. Categorization: Aesthetic — Improves facial appearance and symmetry. 5. Eyelid Reconstruction (Canthoplasty) Procedure: Surgery to correct the down-slanting eyes and repair missing or malformed lower eyelids. Procedure details: Surgeons tighten or reposition the canthal tendons and may reconstruct the eyelid tissues for improved function and appearance. Categorization: Aesthetic — Restores eyelid function and improves the cosmetic appearance of the eyes. 6. Scar Revision Surgery Procedure: Following reconstructive surgeries, scar revision may be performed to reduce visible scars and enhance aesthetic outcomes. Procedure details: Techniques such as laser therapy or surgical excision are used to minimize scarring. Categorization: Aesthetic — Focuses on improving the appearance of surgical scars.		
**Tricho-dento-osseous Syndrome**	CFS	Bn	Clinical evaluatio	*DLX3*	Impact: hair, teeth, bone abnormalities,	0.00001 000	Generall y normal with	Surgical correction of	bones. It is characterized by thickened cranial	1. Dental Reconstruction (for Hypoplastic Enamel and Taurodontism)	Mixed	(304-307)

			n, genetic testing		requires supportive care		proper manage ment	craniofacial anomalies	bones, enamel hypoplasia, and kinky hair.<br><br>Craniofacial Involvement:<br><br>Bone Defects: Craniofacial bone defects include thickened and dense cranial bones, which may lead to a prominent forehead and facial asymmetry. Dental anomalies, such as enamel hypoplasia and taurodontism (enlarged tooth pulp), are also common. Cartilage Defects: There are no specific cartilage defects associated with TDO. Plastic Surgery Correction: Plastic surgery may be required to address craniofacial bone asymmetry. Dental treatment, including orthodontics and possibly reconstructive dental surgery, is often needed to manage the dental anomalies.	Procedure: Dental restorations or crowns are used to protect and restore the function of teeth affected by hypoplastic enamel and taurodontism. Procedure details: Crowns, veneers, or dental implants may be required to restore normal tooth appearance and function. Categorization: Functional and Aesthetic — Protects teeth from decay and restores dental appearance. 2. Craniofacial Reconstruction (If Significant Facial Asymmetry) Procedure: Surgery may be performed to correct facial asymmetry caused by abnormal bone growth or defects in the craniofacial structures. Procedure details: Bone grafting or orthognathic surgery may be performed to restore normal facial proportions and improve chewing function. Categorization: Aesthetic and Functional — Restores facial symmetry and function. 3. Orthognathic Surgery (Jaw Surgery) Procedure: For patients with significant jaw misalignment, orthognathic surgery can be performed to realign the upper and lower jaws, improving both aesthetics and function. Procedure details: Jaw repositioning or bone grafting may be used to correct jaw structure. Categorization: Functional and Aesthetic — Improves bite, speech, and facial		

										appearance.<br>4. Hair Restoration Surgery<br>Procedure: In severe cases of hair loss or brittle hair, hair transplantation or scalp treatments may be considered.<br>Procedure details: Hair transplantation may be used to restore hair density.<br>Categorization: Aesthetic — Enhances cosmetic appearance of the hair.		
**Van der Woude Syndrome**	CFS	Bn	Congenital/ present at birth	*IRF6, GRHL3*	Life expectancy generally normal. Quality of life usually good after treatment although affected individuals may experience feeding difficulties, speech/language delays, dental abnormalities, hearing issues relate to cleft palate, and psychosocial challenges from craniofacial differences.	.000029	Rare but the most common cause of cleft lip and palate resulting from genetic variation	Management is multidisciplinary and symptom-directed. Treatment may include cleft lip repair, cleft palate repair, speech therapy, feeding support, orthodontics/ dental care, hearing evaluation, and genetic counseling. Lip pits may also be surgically removed if symptomatic or cosmetically concerning.	Yes, major involvement	Cleft lip repair, cleft palate repair, lip pit excision, secondary craniofacial revisions, alveolar bone grafting, rhinoplasty, speech-related surgical procedures if velopharyngeal insufficiency occurs	Mixed	(308)
**Vascular Malformations (Hypoplastic Internal Carotid Artery)**	PT	N	Imaging studies (CT, MRI)	*Unknown*	Impact: depends on location and size, requires surgical correction	0.00001000	Variable, depends on severity of	Surgical correction of vascular malformations	Not typically involved	Plastic surgery is generally not directly involved in the management of vascular malformations such as hypoplastic ICA.	Neither	(4, 69, 74, 84, 204, 251, 252, 309)

							malformation						
**Von Hippel-Lindau Syndrome**	PT	N	Clinical evaluation, genetic testing	*VHL 22q11.2 deletion, CCND1 (modifier of phenotype)*	Impact: tumors in multiple organs, requires multidisciplinary care	0.00002778	Reduced, variable depending on severity of symptoms	Surgical removal of tumors, supportive care	Not typically involved	While plastic surgery is not directly involved in the management of tumors associated with VHL	Neither	(256, 281, 310-312)
**Yemenite deaf-blind hypopigmentation syndrome**	CO	N	congenital	*AR, no well characterized mutations*	significant decline in QOL due to sensory dfeicits, no changes in LE	0.00000100	Prevalence: Extremely rare, with fewer than 50 documented cases, primarily in the Yemenite Jewish population	supportive care	none	none	Neither	(42, 313)
